# Collapse of the Helical Hydrogen Bond Structure in Imidazolium Hydrogen *o*‑Phthalate Under High Pressure: A Vibrational Spectroscopy Study

**DOI:** 10.1021/acs.jpclett.6c01203

**Published:** 2026-04-28

**Authors:** Sylwia Zięba, Christelle Kadlec, Adam Mizera, Elena Buixaderas, Miroslav Lebeda, Petr Ondrejkovič, Jirka Hlinka, Petr Kužel

**Affiliations:** 1 Institute of Physics, Czech Academy of Sciences, Na Slovance 1999/2, 182 00 Prague 8, Czech Republic; 2 119441 Institute of Molecular Physics, Polish Academy of Sciences, M. Smoluchowskiego 17, 60-179 Poznan, Poland; 3 Faculty of Mechanical Engineering, Czech Technical University in Prague, Technická 4, 166 07 Prague 6, Czech Republic

## Abstract

Imidazolium hydrogen *o*-phthalate (OrtImi) is an organic ionic crystal with a helical hydrogen-bonded network. We report on the evolution of the Raman, terahertz, and infrared modes of OrtImi over a wide range of temperatures and hydrostatic pressures. We interpret the results using density functional theory and molecular dynamics within machine-learning interatomic potential framework calculations. The vibrational spectra uncovered a phase transition occurring above 3 GPa applied hydrostatic pressure. This transition is associated with a collapse of the helical network of N^+^–H···O^–^ hydrogen bonds transforming into a ring motif within the *ab* plane. Proton ordering in hydrogen bonds emerges upon cooling below 150 K, creating favorable conditions for proton–phonon coupling.

## Graphical abstract

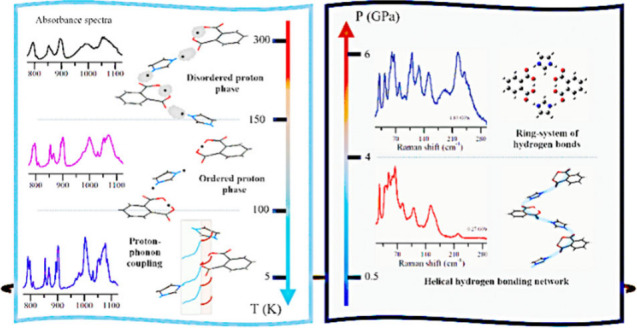

Hydrogen bonds (HBs) are crucial for the molecular arrangement of organic crystals.^1,2^ They play an essential role in the physical properties of materials such as structural flexibility,^3–5^ and their macroscopic properties, namely the thermal stability and thermal and electrical conductivity, depend on their type, strength, and number.^6–8^ Assessing the evolution of HB networks upon changing external parameters is important for understanding the physical properties of materials such as proton conductors^1,7^ or energetic materials.^9^

Hydrogen-bonded organic frameworks (HOFs) represent a novel class of porous systems with unique dynamical properties that have attracted considerable interest in view of diverse applications such as optical or information storage.^10,11^ These materials are composed of organic molecules or ions that are linked by HBs, which determine their behavior under different thermodynamic conditions which are defined, e.g., by the temperature or pressure.^12^ Uncovering the underlying mechanisms is then crucial for designing new, more efficient organic materials.^1,13^ Organic compounds, such as imidazole, pyrazole, and triazole, are used as building blocks for such systems due to their environmentally friendly properties.^1,12^

Recent studies have been conducted to determine the proton dynamics mechanisms in proton conductors with HBs.^14,15^ Among other factors, proton mobility in such compounds is influenced by the type of HB network (1D, 2D, or 3D), the number of HBs, and their energy.^15–18^ It has been proposed that chemical engineering of supramolecular clusters within HOF structures may lead to the forming of proton hubs enabling efficient proton transport.^14^ Such a strategy promises to overcome the current limitations of proton conduction along one-dimensional chains linked by HBs. Pressure, alongside humidity and temperature, is a thermodynamic parameter that allows HB networks to be manipulated.^15^ Understanding the nature of HBs and how they change with pressure is crucial not only for the conductive properties in HOFs but also in metal–organic and covalent organic frameworks.^19^

In this context, our study provides new insights into the structure–property relationships of supramolecular materials under high pressure and offers valuable perspectives for developing pressure-sensitive switching materials. The phase transition reported here is the first one observed in an organic salt with a helical hydrogen-bonding structure.

Understanding the physical processes occurring in such materials under temperature or pressure changes requires an understanding of the proton-lattice and of the proton-phonon coupling.^7,20^ As Huang et al. (2023) suggested,^1^ an efficient proton transfer can occur if associated with phonons.^21^ The change in phonon frequencies due to a change in volume or pressure at a fixed temperature is known as an implicit anharmonicity.^3^ In fact, the OH and NH stretch regions in vibrational spectra contain rich information about the anharmonicities and about the dynamics of HBs and their microscopic environments,^22^ which can be advantageously investigated experimentally using the vibrational spectroscopy.^6,7,9,20,21^ Changes in HBs within molecules can then be spectroscopically detected without measuring their length.^23^ Applying mechanical pressure provides an alternative method of inducing significant changes in structural, optical, and electronic behaviors, including fundamental properties rarely observed under ambient conditions.^24,25^

Imidazolium hydrogen *o*-phthalate (OrtImi) is a molecular system in which ions are connected by N^+^–H···O^–^ HBs and form a unique helical structure under ambient conditions. It is also a proton conductor with a maximum specific electrical conductivity value of 10^–3^ S/cm.^26^ A weak van der Waals force exists between layers of benzene and imidazole rings, resulting in compact molecular packing and a stable crystal structure. This paper presents an analysis of changes in the arrangement of ions connected by HBs, as observed using Raman, THz, and IR spectroscopies at low temperatures (5–300 K) and high pressures (up to 7 GPa). The changes in the parameters of the most important bands for the physics of the material are directly shown and discussed in the paper; for the completeness of the study and to support further our interpretation we provide an extensive number of complementary results in the Supporting Information. The analysis of the observed changes in the studied system was carried out by involving quantum chemistry calculations, which were performed using density functional theory (DFT) and molecular dynamics within the machine-learning interatomic potential framework methods.

The helical structure of N^+^–H···O^–^ HBs in OrtImi is presented in Figure 1 and Figure S1a. A similar helical shape of the hydrogen-bond network is observed in BenImi,^2^ SalImi,^27^ and TerImi.^28^ However, in contrast with those materials, an intramolecular O···H···O^–^ HB present in OrtImi appears as a significant part of the helix structure (see Figure 1). This structure is essential for understanding how the studied crystal behaves at different temperatures and pressures.

**Figure 1:**
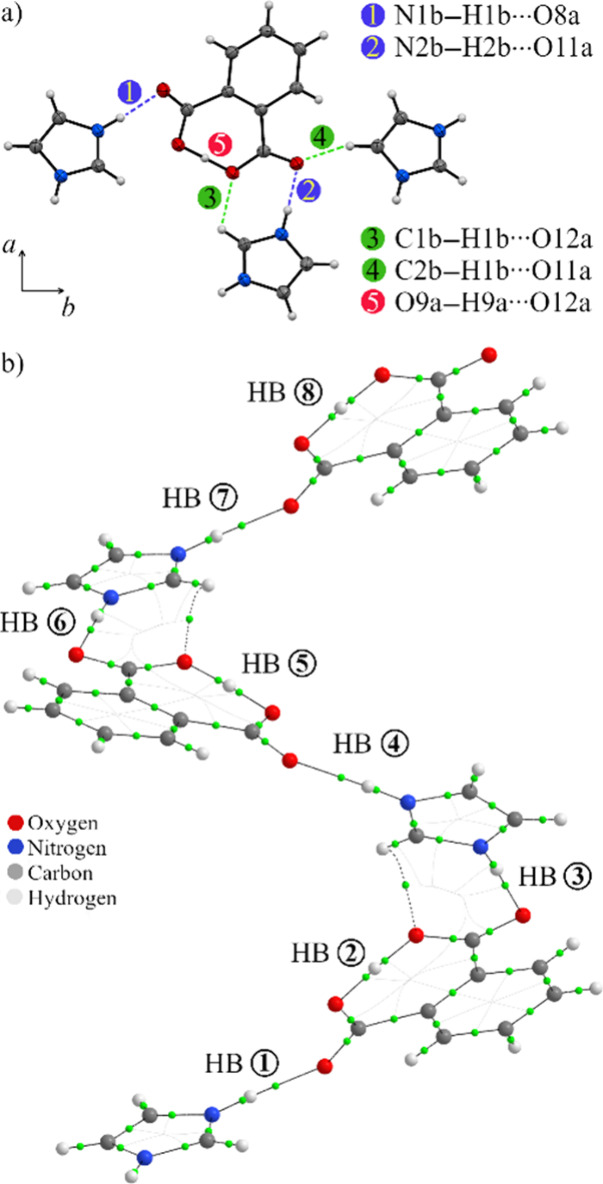
A view of the molecular ions of OrtImi showing inter- and intramolecular HBs (a). Representation of bonding interactions in helical structure (b). Note: green circles in (b) correspond to the bond critical point (BCP).^29^ Structures a and b were prepared using the crystal structure deposited at the Cambridge Crystallographic Data Centre (CCDC ID: 1847597).^26^ Non-H atoms are depicted as 30% probability displacement ellipsoids.

We first describe the helical structure containing N^+^–H···O^–^ and O···H···O^–^ HBs in OrtImi. The basic characteristics of the previously published OrtImi crystal structure^26^ and our DFT theoretical analyses of the HB networks by means of the PES and the Quantum Theory of Atoms in Molecules (QTAiM) are presented in the Supporting Information (Description S1). Theoretical computations were carried out at the DFT/ωB97x-D/6–311G++(d,p) level of theory, which is proper for compounds with HBs.^30^ The helix is characterized by an ellipse in the *ab* plane with the major half axis *a*_h_ = 4.21 Å, the minor half axis *b*_h_ = 3.86 Å and by the pitch Sh_h_ = 11.35 Å (Figure S2). Based on the energy and topological parameters^31^ (Tab. S1) N^+^–H···O^–^, O···H···O^–^, and C–H···O HBs are medium, strong, and weak, respectively.

The helical structure of OrtImi consists of three interlaced helicoids (see Figure S3). All three helicoids are formed along a single axis in the [001] direction (Description S2 and Table S2). The N^+^–H···O^–^ HBs can be described using an asymmetric potential well with two energy minima (see Figures S4 and S5), whereas O···H···O^–^ HBs are characterized by a symmetric potential energy with one minimum (Figure S5a).

Infrared absorbance, THz, and Raman spectra were measured to analyze the vibrational properties of OrtImi (see Descriptions S3 and S4, Figures S6–S8). The measured spectra were compared to the theoretical calculations made using DFT methods to assign the observed bands (Description S5, Figures S9–S13). The molecular arrangement, for which these calculations were performed, is shown in Figure S11.

First, temperature-dependent bands for O···H···O^–^ HBs were analyzed. The IR band at 1058 cm^–1^, assigned to the γ­(OHO^–^) mode, shifts toward a higher wavenumber as the temperature decreases (blue shift, Figure 2a). Due to its anharmonic shape (see Figure S14a), the band was fitted using the SplitPearson7 function in the Fityk program.^32^ Figure 2 shows also other important parameters of this vibration mode issued from the fit, namely the anharmonic factor Ψ­(*A*), the full-width-at-half-maximum Δν (FWHM) and the parameters *a* and *b* characterizing the low-frequency half-width-at-half-maximum and high-frequency, respectively (i.e., Δν = *a* + *b*; see also Figure S15). We plot here also the damping parameter Δν/ν_0_ (where ν_0_ is the band position), and the maximum energy absorption *F*[*A*_C_(ν_0_)] calculated from the fit parameters. The anharmonic factor is calculated based on asymmetry of the energy absorption curve using equations: Ψ­(*A*) = 1 + $\left(\frac{1}{\pi }\right)\left[1-\frac{b}{a}\right]$ for *a* > *b* and Ψ­(*A*) = 1 – $\left(\frac{1}{\pi }\right)\left[1-\frac{a}{b}\right]$ for *b* > *a*.^33^ Its value for the discussed band shows a weak temperature dependence between 300 and 150 K: the value is slightly smaller than 1, and we found that *b* > *a* indicates a soft anharmonic force (due to overlapping of outer electron shells). Below 150 K, larger changes in several fit parameters are observed. We namely observe a crossing at 150 K in the temperature evolution of *a* and *b*; this drives the anharmonic factor to a hard characteristic and the nature of the band changes to a hard anharmonic force (only deformation of the electron cloud is caused by the vibration) below 150 K. The overall decrease in the damping coefficient levels off below 150 K (Figure 2d).

**Figure 2:**
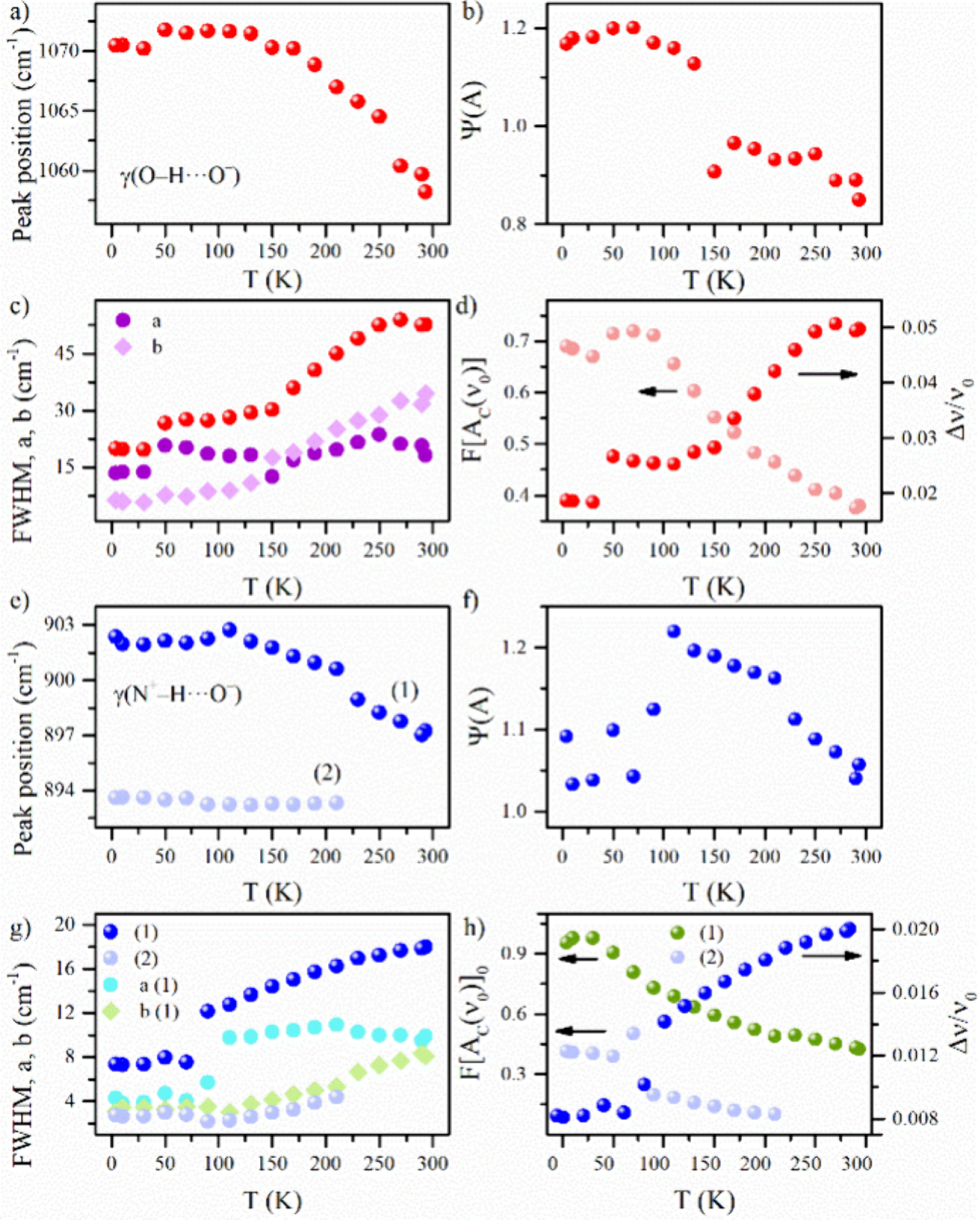
Anharmonicity analysis of selected IR bands of OrtImi as a function of temperature (from spectra in Figure S14): Peak position *v*_0_ (a, e), anharmonic factor Ψ­(*A*) (b, f), FWHM and the high- and low-frequency *a* and *b* (c, g), and damping parameter Δν/ν_0_ and maximum energy absorption *F*[*A*_C_(ν_0_)] (d, h). Bands connected to γ­(O···H···O^–^) are depicted in the upper part (a–d) and those connected to γ­(N^+^–H···O^–^) are shown in the lower part (e–h).

Analysis of the anharmonicity of the IR band at 2982 cm^–1^ did not reveal any significant changes in its thermal behavior in the 300–5 K range (Figures S15a and S16a–d). To determine the effect of temperature on the O···H···O^–^ bond length, the position of the IR band at 1924 cm^–1^ was also analyzed (see Figure S17a): we observe here a red shift upon cooling. The calculated IR spectra show that this shift is related to a shortening of the *d*_O···O_ distance and an elongation of the O–H bond as the temperature decreases from 300 to 130 K (see Figures S10 and S12). Below 130 K, the position of this band remains unchanged (Figure S17). Within the *d*_O···O_ distance range of 2.3–2.5 Å, the HB energy decreases dramatically from approximately −105 to −40 kcal·mol^–1^ (Figure S18). In such a case, the HB angle is close to 180°, meaning that the character of the bond is strongly covalent. At a bond length of 2.33 Å, the energies of the two O–H bonds in the O···H···O^–^ HB are the same, indicating a high symmetry and a high strength of the HB. In this case, it is characterized by a symmetric potential well with a single energy minimum (Figure S19). When the temperature is lowered, the band at 1924 cm^–1^ becomes more intense and also visible in the FTIR spectra (Figure S14b). This effect is associated with a decrease in hydrogen dynamics of the bond as the temperature decreases.^34^

Similar analysis was done for N^+^–H···O^–^ HBs which create a helical system. As the temperature decreases, each of the IR bands at 794, 853, 896, 1434, and 1455 cm^–1^ connected with N^+^–H···O^–^ HBs splits into two components (Figure S14). In Figure 2e–h we plot the temperature evolution of the 896 cm^–1^ band as an example. Since the band has an asymmetric shape, it was fitted with an anharmonic function, as described above for the γ­(OHO^–^) band. A blue shift of this band is observed as the temperature decreases from 300 to 100 K; in the 100–5 K range, the band position remains unchanged. We also observed the appearance of the split component at 220 K (Figure 2e). This component also slightly shifts toward larger wavenumbers as temperature decreases.

The anharmonic factor increases from approximately 1.03 to 1.23 as the temperature decreases from 300 to 100 K, indicating that the band becomes increasingly anharmonic within this range. The half-width of both split bands decreases in this temperature range (Figure 2g), and the damping parameter decreases as well. All anomalies in the behavior of the anharmonic parameters of the 897 cm^–1^ band are observed at 100 K. Below 100 K, the value of the anharmonic parameter sharply steps down to approximately 1.1 (Figure 2f), indicating a mostly harmonic nature of this band. Furthermore, the half-width of the bands and the a and b parameters keep an approximately constant value.

Anharmonicity analysis was also carried out for the IR band at 3099 cm^–1^, which is associated with the stretching of the N^+^–H···O^–^ HBs (Figure S16e–h). No anomalies were observed in the temperature behavior of these band parameters. In the temperature-dependent Raman spectra, no significant anomalies or mode splitting was observed (see Figure S20). Only two new weak bands appeared at 855 and 864 cm^–1^ below 100 K.

For proton dynamics in a helical system, a deep insight into lattice vibrations is necessary. In the THz range, a shift of the bands toward a higher wavenumber on cooling is evidenced (Figure 3a). Below 100 K, we observe a slight change in the slope of the dependence of the band position on temperature (Figure S21). The position of the ν_2–4_ bands as well as their FWHM remain approximately constant below 100 K (Figure S21a–i). The parameters of the ν_6,7_ bands change below 100 K (Figure S21j–o). This is because these bands are primarily associated with the dynamics of the N^+^–H···O^–^ HBs network (see Table S3). The temperature evolution of the Raman spectra shows splitting of the ν_9_ (109 cm^–1^) band into two components below 150 K (Figure 3c,d). The intensity and half-width of the ν_10_ and ν_11_ bands do not change significantly in the 300–150 K range (Figure S22). Below 150 K, however, we observe a significant increase in their intensities and a decrease in their FWHM.

**Figure 3:**
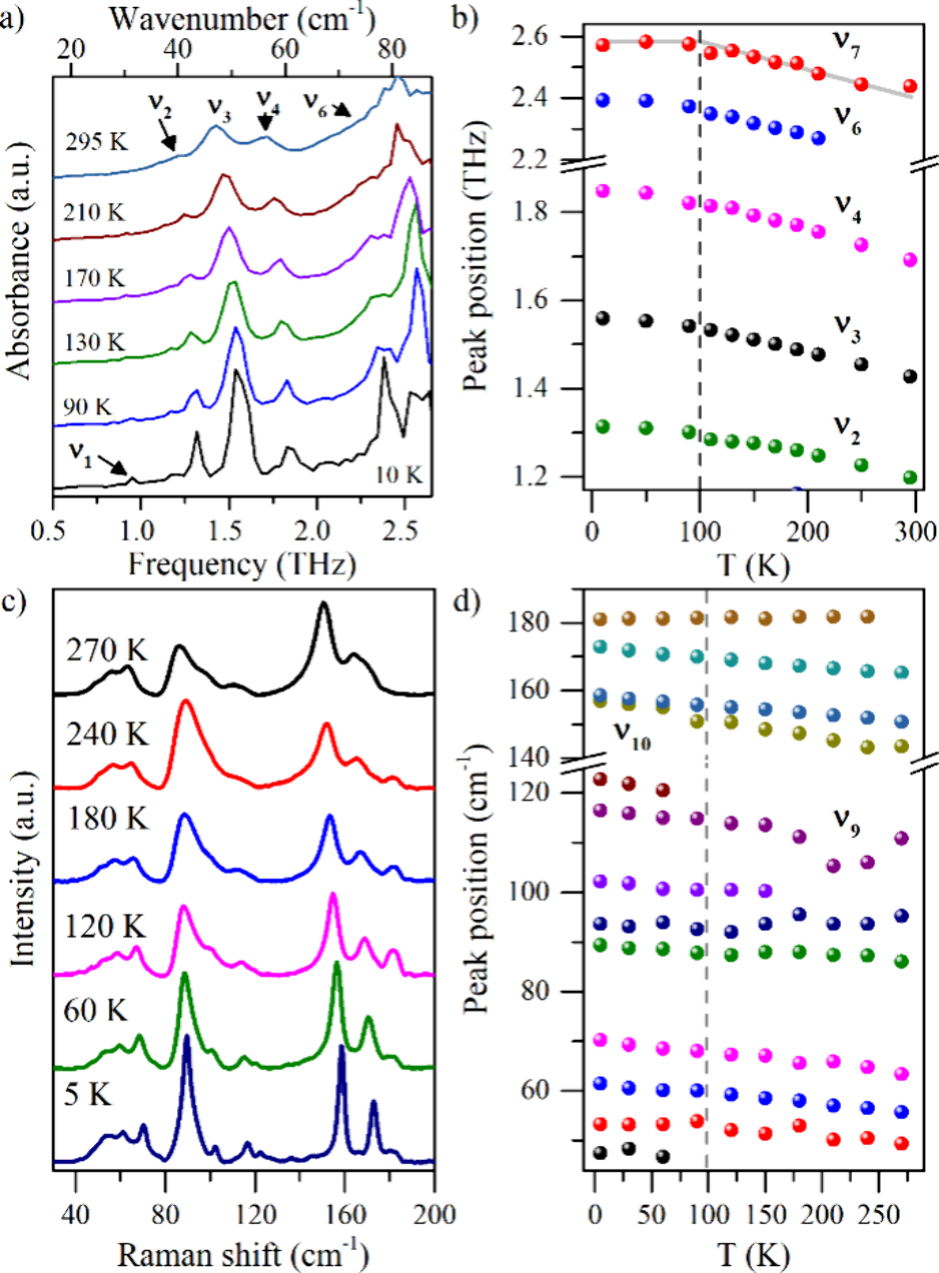
THz and Raman spectra of OrtImi as a function of temperature (a, c), respectively. Temperature dependence of the position of THz peaks (b) and Raman bands (d).

The temperature-dependent spectroscopic analysis revealed two distinct temperatures at which significant changes occur in OrtImi: 150 and 100 K. These changes are related to the helical network of N^+^–H···O^–^ and O···H···O^–^ HBs. In the 300–150 K range, the dynamics of OrtImi are predominantly influenced by the stretching vibrations of the strong O···H···O^–^ HBs. This is due to the nature of strong HBs, which have a symmetric potential well where the position of the hydrogen atom is blurred between the donor and acceptor due to strong anharmonic coupling. Below 150 K, the position of hydrogen in this bond becomes ordered (takes a well-defined position in HB), as evidenced by the change in the intensity of the IR bands at 992 and 1058 cm^–1^ (see Figure S14a). Additionally, the half-width of these bands decreases by approximately 18 cm^–1^ in the 300–150 K range.

Below 100 K, the HBs become ordered, as evidenced by the half-width remaining constant within this temperature range (Figure 2c). Furthermore, numerous mode splittings are observed in the bands associated with N^+^–H···O^–^ HBs deformation vibrations. As, in general, splitting does not occur for longitudinal vibrations, our finding suggests that the vibrations in these bonds couple transversely. This coupling is assisted by the dynamics of the proton in the O···H···O^–^ HB (proton-phonon coupling). This is supported by the similar behavior of the bands associated with the phonon vibrations of the N^+^–H···O^–^ and O···H···O^–^ bridges: indeed, both the bands associated with N^+^–H···O^–^ deformation vibrations and those associated with the tensile vibrations of O···H···O^–^ are split. In OrtImi, the O···H···O^–^ HB is part of the helix. For this reason, it significantly impacts the coupling of N^+^–H···O^–^ HBs. This is in contrast with SalImi, where O–H···O HBs also occur, but they are not part of the helical system and do not contribute to the coupling of HBs.^27^

Pressure has a great effect on the position and orientation of molecules within the helical HB network of OrtImi. Under increased pressure, some vibration modes undergo blue shifts due to the increased intermolecular force and decreased molecular spacing.^12^ This can be seen when analyzing the IR bands associated with C–H bond dynamics in the 3120–3250 cm^–1^ range (Figure S23). While there is no major change in the parameters of ν­(C–H) bands with temperature (Figure S23a,c,e,f) these bands blue shift by approximately 50 cm^–1^ with increasing pressure (Figure S23b), suggesting a gradual shortening of the C–H···O HBs under pressure. Significant changes in the surface area, intensity, and half-width of these bands are observed mainly at 3 GPa (Figure S23d,e,g). These observations suggest that, up to 3 GPa, the ions get closer to each other without any structural change. A pressure higher than 3 GPa introduces additional changes to the position and orientation of ions within the crystal lattice, but it also introduces changes in the internal structure of ions.

To understand the proton dynamics under pressure the analysis of bands characteristic for O···H···O^–^ HBs was done. The IR bands at 2982 and 1058 cm^–1^ shift by approximately 40 cm^–1^ toward higher wavenumbers as pressure increases (blue shift, Figure 4a, Figures S24a and S25). A nonmonotonic pressure dependence of the anharmonicity coefficient for the γ­(O···H···O^–^) + ν­(O–H) mode at 2982 cm^–1^ occurs and it presents a minimum around 3 GPa. At higher pressures, the coefficient increases toward 1, i.e., toward mostly harmonic motion: the values of *a* and *b* approach each other above 4 GPa (Figure 4b–d). In the case of the band parameters of γ­(O···H···O^–^) at 1058 cm^–1^, we do not observe any major changes in the analyzed pressure range (above 4 GPa, Figure S26b–d). Slight changes in the behavior of the bands suggest that, at a pressure of above 4 GPa, a phase transition occurred throughout the entire volume of the crystal, which is now at its minimum energy.

**Figure 4:**
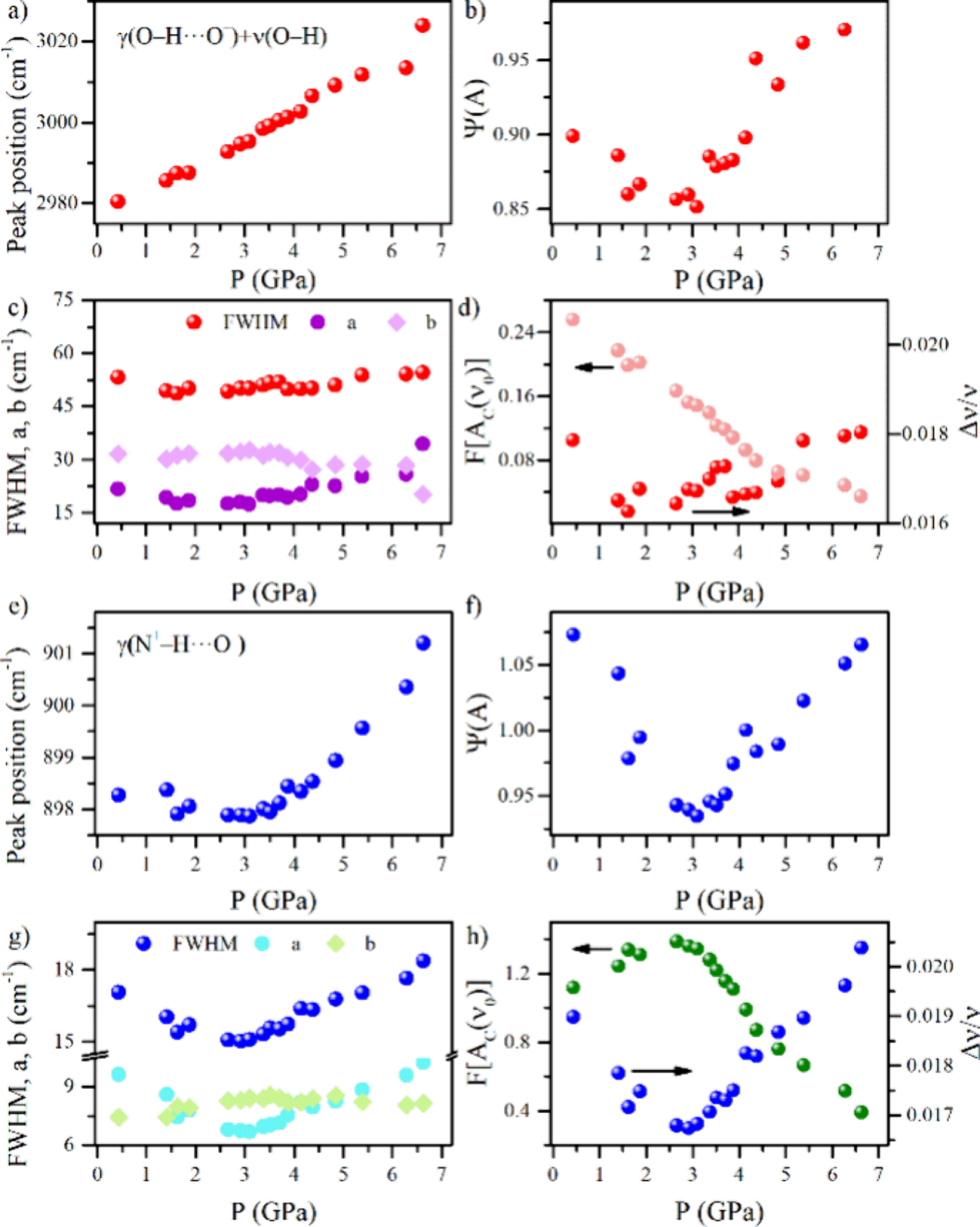
Anharmonicity analysis of selected IR bands of OrtImi as a function of pressure (from spectra in Figures S24 and S25): Peak position *v*_0_ (a, e), anharmonic factor Ψ­(*A*) (b, f), FWHM and the high- and low-frequency *a* and *b* (c, g), and damping parameter Δν/ν_0_ and maximum energy absorption *F*[*A*_C_(ν_0_)] (d, h). Bands connected to γ­(O···H···O^–^) + ν­(O–H) are depicted in the upper part (a–d) and those connected to γ­(N^+^–H···O^–^) are shown in the lower part (e–h).

In the pressure range from 0.13 to about 1.50 GPa, the O···H···O^–^ HBs shorten (the position of the 1920 cm^–1^ band red shifts; Figure S17b). Our analysis indicates that the greatest changes occur in the direction longitudinal to the O···H···O^–^ HBs from about 3–4.4 GPa. The intermediate pressure range 1.5–3.0 GPa appears to be transitional, with no major changes occurring in the system.

To facilitate a better understanding of the observed changes, bands connected to N^+^–H···O^–^ HBs were analyzed. The IR band at 3099 cm^–1^, assigned to the ν­(N–H) mode, blue shifts up to a pressure of about 4.6 GPa, (Figure S26e): this corresponds to an elongation of the N^+^–H···O^–^ HB, which is accompanied by a shortening of the N–H bond. Subsequently, at 4.6 GPa, its frequency exhibits an abrupt step down by 0.5%; this jump in frequency is followed by another blue shift upon the pressure increase. In the case of the ν­(N–H) band parameters, we do not observe any major changes in the analyzed pressure range.

The frequency of the IR band at 896 cm^–1^, assigned to γ­(N^+^–H···O^–^), starts to increase at about 3 GPa (see Figure 4e and Figure S24). The pressure dependencies of the anharmonicity coefficient, half-width, and damping coefficient also change at 3 GPa (see Figure 4f–h). The damping coefficient decreases to about 3 GPa, after which it increases (Figure 4h). These results suggest that, up to approximately 3 GPa, the intermolecular distances affecting the orientation and arrangement of ions decrease and the system tends toward the energy minimum. At pressures above 3 GPa, in addition to changing the orientation and arrangement of ions, modifications are introduced to the internal structure of ions (the damping coefficient increases). Since the shape of the analyzed band changes as the pressure increases, the nature of the interactions in the molecular system must change as well.

Above 2.5 GPa, the 896 cm^–1^ Raman band splits into two components (Figures S27 and S28). A similar splitting of the band associated with N^+^–H···O^–^ HBs was observed for SalImi when a mutual coupling of N^+^–H···O^–^ HBs was observed.^27^ An additional Raman band appears at approximately 625 cm^–1^ (Figure S28). We can also observe a change in the position of three bands at 1134, 1145, and 1167 cm^–1^ in the pressure evolution of Raman spectra (see Figures S29–S32). As the pressure increases, we observe a blue shift in each of these bands but with different dynamics: up to 4.8 GPa they shift by 23, 29, and 11 cm^–1^, respectively. The band at 1145 cm^–1^ shifts more drastically than the others. A very slight shift in the 810 and 1107 cm^–1^ bands (Figures S28–S30), which are associated with the benzene ring, indicates that the benzene ring is not deformed.

To understand the dynamics of the hydrogen-bond network in relation to pressure, the lattice vibrations were analyzed. Between 0.13 and 1.5 GPa of pressure (Figure 5a), the most intense bands are found in the Z1 range, indicating that the lattice exhibits the strongest dynamics in the direction transverse to the helical system. Above 1.5 GPa, the number of phonon vibrations changes. An additional band appears at 27 cm^–1^ and another band disappears at 54 cm^–1^ (Figure S33b). The bands in the Z2 range become more intense with increasing pressure, and the number of components also changes (Figure 5a). The ratio of the intensities of the 149 and 160 cm^–1^ bands also changes (Figure S34). Above 1.5 GPa, the 160 cm^–1^ band becomes more intense than the 149 cm^–1^ band and its frequency progressively increases with increasing pressure up to 215 cm^–1^ (i.e., by 55 cm^–1^ compared to the atmospheric pressure). The changes indicate that the pressure increase from 1 to 4 GPa deeply modifies the helical HB network.

**Figure 5:**
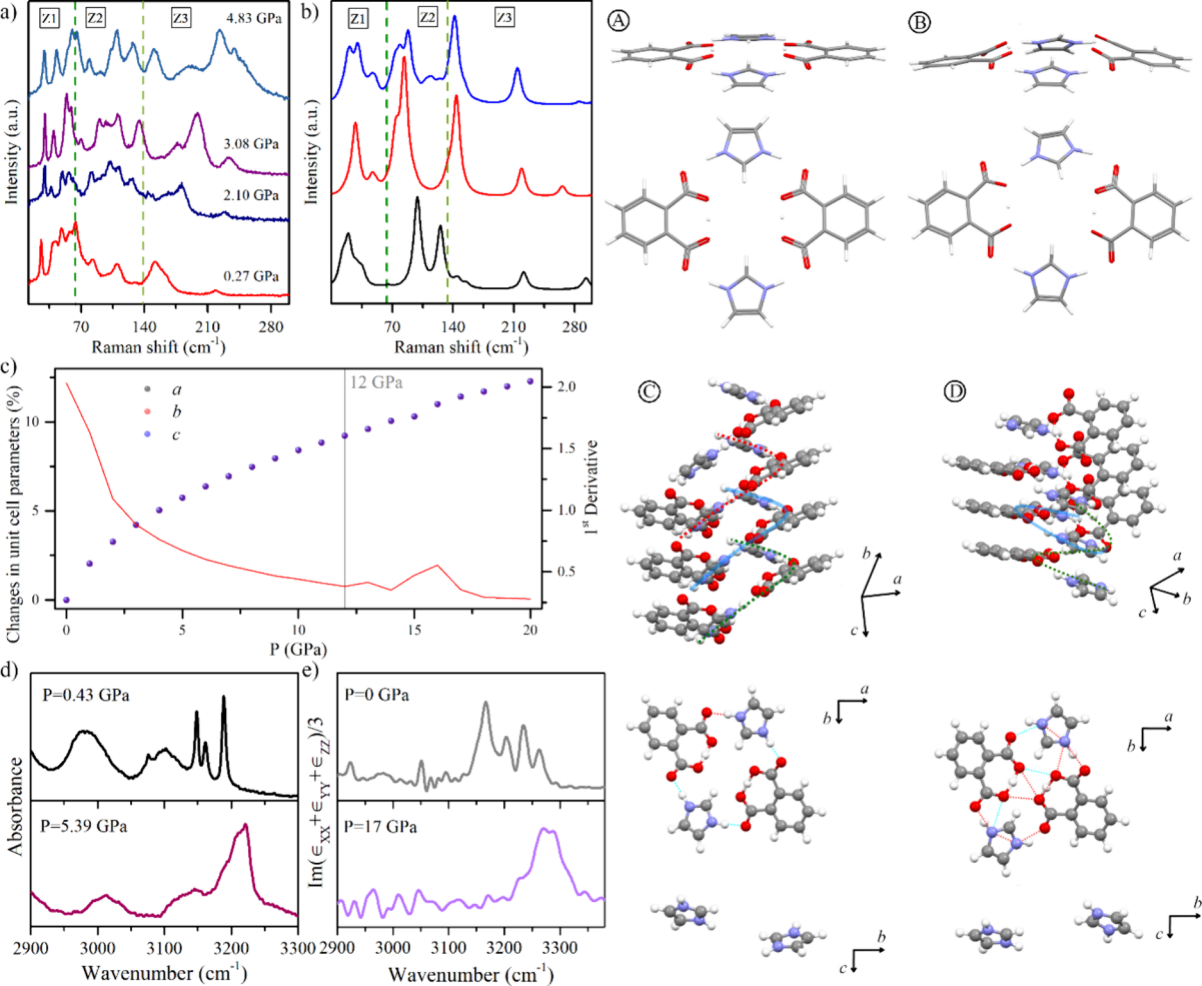
(a) Raman spectra measured for selected pressures: 0.27, 2.10, 3.08, and 4.83 GPa. (b) Calculated Raman spectra for three different molecular arrangements: the arrangement of three ions (with *d*_O···O_ = 2.38 Å, which corresponds to the distance obtained from X-ray measurement at atmospheric pressure; see Figure S11, black line). The A-ring system is optimized from a helical arrangement (red), and the B-ring system is optimized from a ring arrangement (blue). Percentage changes in the elementary cell parameters as a function of pressure and the first derivative of these changes (red line, c): comparison of experimental at 0.43 and 5.39 GPa (d) and calculated at 0 and 17 GPa (e) FTIR spectra. Arrangement of ions in the calculated OrtImi crystallographic structure, as determined by molecular dynamics within the machine-learning interatomic potential framework (uMLIP MACE-MH-1 with dispersion correction) at: 0 GPa (C) and 17 GPa (D).

We performed DFT calculations to simulate the most probable molecular arrangement when reducing the distance between helices (more details in Description S6). We used these calculations to model a system of three ions (with *d*_O···O_ = 2.38 Å, which corresponds to the distance obtained from X-ray measurements;^26^ see Figure S11) corresponding to a fragment of a helix. This shows the most probable spatial distribution of imidazole ions in relation to the acid ion at atmospheric pressure.

When a system of HBs is structurally completely symmetrical, the whole system exhibits a high degree of proton polarizability due to the strong mutual coupling of proton motions.^21^ Above 3 GPa, the ion rings have moved significantly closer to each other, resulting in a decrease in the dynamics of transverse vibrations. The increase in the intensity of the bands associated with the stretching of the N^+^–H···O^–^ and O···H···O^–^ HBs suggests that longitudinal phonons begin to play an important role in the lattice dynamics, similar to that of transverse phonons. Comparing the calculated spectra for systems A and B indicates that the number of components in the B spectrum (Figure 5a,b) agrees with the number of observed modes in the experimental spectrum at 4.83 GPa.

To confirm a pressure-induced phase transformation in OrtImi, molecular dynamics simulations were performed using the universal MLIP of MACE-MH-1 with the omat_pbe head and D3 dispersion corrections (more details are given in Description S7). Analysis of the unit cell parameters calculated using molecular dynamics in the 0–20 GPa pressure range for OrtImi showed that the structure is stable up to 12 GPa (Figure 5c). Above 12 GPa, a change is observed, which is particularly visible when the first derivative is plotted (Figure 5c). The main change occurs between 15 and 16 GPa. In the calculations, the volume of the unit cell increases by 25% at 12 GPa. For comparison, experimentally determined changes in volume for similar salt crystal structures were 13% at 2.27 GPa (SalImi, *V* = 1972 Å^3^)^27^ and 17% at 3.41 GPa (BenImi, *V* = 928 Å^3^).^2^ Given the much larger unit cell (for OrtImi *V* = 4089 Å^3^) and a higher pressure of the experimental phase transition, it seems that a pressure of 12–17 GPa obtained by modeling may reflect the changes observed experimentally within the 4–6 GPa range. Analysis of the calculated crystal structure showed that the imidazole ion arrangement changes as a result of the phase transition. At 17 GPa, two N^+^–H···O^–^ HBs with donor–acceptor lengths of 2.68 and 2.59 Å are formed between imidazole and two acid sites belonging originally to two different helical systems. As a consequence, a ring motif of N^+^–H···O^–^ HBs sets in within the *ab* plane (see Figure 5Ⓒ,Ⓓ arrangements).

To further confirm the observed phase transition, FTIR spectra were calculated for crystal structures obtained at pressures ranging from 0 to 20 GPa (see Figure S35). The calculated spectrum shows a similar number of components compared with the experimental spectrum. The ν_COO_ band can be distinguished slightly above 1400 cm^–1^, shifting toward a higher wavenumber with increasing pressure (Figure S36), in a similar manner to that observed close to 1400 cm^–1^ in the experiment. In the 2800–3500 cm^–1^ range (see Figure 5e and Figure S35b), four bands associated with the stretching of CH and NH bonds are observed at low pressures. As the bond motif changes from helical to planar, a similar intensive broad band appears in both the experimental and theoretical spectra (Figure 5d,e).

The calculated unit-cell volumes per formula unit of each phase have been fitted by the third-order Birch–Murnaghan (BM) equation of state (BM-EOS).^35,36^ The obtained bulk modulus *B* and its pressure derivative *B*′ equal *B* = 16.3 ± 0.4 GPa and *B*′ = 7.1 ± 0.2 (for Phase I) and *B* = 49.5 ± 28.2 GPa and *B*′ = 2.4 ± 1.3 (for Phase II). In Phase I, OrtImi exhibits a high compressibility, similar to CH_3_NH_3_PbBr_3_ ^16^ or NH_2_Cl^37^ and 2-amino-6-nitrobenzothiazole.^38^ Above the phase transition, the compressibility of the system significantly decreases. In comparison, the structure of imidazolium salicylate (SalImi) is lacking long-range order at a pressure of approximately 2.5 GPa, which is indicative of its superior compressibility to OrtImi, as evidenced by the lower value of this coefficient (for SalImi *B* = 14.5 GPa).^27^ The high value of the parameter *B*′ in OrtImi (7.1) indicates an unusual, e.g., anharmonic, behavior, since a harmonic motion typically implies *B*′ ≈ 4.^35^

The experimental results together with the theoretical calculations demonstrate that the application of high hydrostatic pressure induces a phase transition in OrtImi, transforming its helical structure into a planar one, where anions and cations are connected in a ring motif by N^+^–H···O^–^ HBs. At atmospheric pressure, the three helices are arranged in identical directions with the same twist (see Figure S3c). As the hydrostatic pressure increases, the distance between ions decreases, as does the distance between helices. At sufficiently high pressure (experimentally, between 3 and 4 GPa), the distance between ions becomes so small that additional HBs form, changing the structure from a helical to ring system and causing a phase transition.

Changes in HB network in organic materials lead to significant changes of the material properties, such as order–disorder transition,^37^ amorphization,^16^ hydrogen bond symmetrization,^17^ symmetry breaking,^16,39^ or conformational isomerization.^39^ In this context, pressure acts as a thermodynamic variable enabling controlled alterations in the HB network, and its application provides an effective method of obtaining new phases in HOFs with potentially suitable properties for use as proton conductors. Therefore, understanding the proton mobility in HOFs is crucial for designing new functional compounds.^15–17^ Our analysis of HBs establishes a key finding: helices supported by strong hydrogen bonding display superior stability under high-pressure conditions relative to analogous compounds lacking such interactions.^2,27^

In summary, using a wide range of spectroscopic methods (THz, IR, Raman) and density functional theory and molecular dynamics computational analysis within the machine-learning interatomic potential framework, we assessed the behavior of vibrational modes of imidazolium hydrogen *o*-phthalate (OrtImi) under variable temperature and hydrostatic pressure and deduced its consequences for the general physical properties of the system. We found that the helical network formed by N^+^–H···O^–^ and O···H···O^–^ hydrogen bonds and its evolution under pressure plays a leading role. Namely, a pressure-induced phase transition above 3 GPa is observed, where the helical HB network changes into a ring structure. Furthermore, we demonstrated that decreasing the temperature results in proton ordering in HBs below 150 K, which makes conditions conducive to proton–phonon coupling favorable. These findings provide a promising basis for developing pressure-sensitive materials with a switchable proton conductivity.

## Supplementary Material



## Data Availability

All calculation files used to perform the analysis described in the paper have been placed in a publicly accessible database: 10.5281/zenodo.18713028.

## Notes

Synthesis procedure and analyses of the HB networks by means of DFT, molecular dynamics, PES, and QTAiM. Description of the crystallographic structure of OrtImi, including its hydrogen bonds (Description S1). DFT calculation of the energy barriers (Description S2). Experimental and theoretical analyses of the band positions (Description S3). Determination of the position of the νO–H band in the strong O···H···O^–^ hydrogen bond (Description S4). Assignment of bands associated with phonon vibrations (Description S5). Analysis of the DFT and molecular dynamics calculation results (Descriptions S6 and S7) (PDF)
