# Supplementary material for: Collapse of the Helical Hydrogen Bond Structure in Imidazolium Hydrogen o‑Phthalate Under High Pressure: A Vibrational Spectroscopy Study
Source: J Phys Chem Lett. 2026 Apr 28;17(18):5266–74. doi: 10.1021/acs.jpclett.6c01203 (PMC13318063; doi:10.1021/acs.jpclett.6c01203)
Supplement: Supplementary file 1 [file jz6c01203_si_001.pdf]

Supplementary information: Collapse of the Helical Hydrogen Bond Structure in Imidazolium Hydrogen *o*-Phthalate Under High Pressure: a Vibrational Spectroscopy Study

Sylwia Zięba[1,2], Christelle Kadlec[1], Adam Mizera[2], Elena Buixaderas[1], Miroslav Lebeda[1,3], Petr Ondrejkovič[1], Jirka Hlinka[1], Petr Kužel[1]

[1]Institute of Physics, Czech Academy of Sciences, Na Slovance 1999/2, 182 00 Prague 8, Czech Republic

[2]Institute of Molecular Physics, Polish Academy of Sciences, M. Smoluchowskiego 17, 60-179 Poznan, Poland

[3]Faculty of Mechanical Engineering, Czech Technical University in Prague, Technická 4, 16607 Prague 6, Czech Republic

Samples and methods

The synthesis procedure of the imidazolium hydrogen *o*-phthalate (OrtImi) was described in reference.[1] Crystals of the phthalic acid and imidazole salt, needle-like crystallites 20 mm in length and about 0.5 mm thick, were obtained.

Scanning electron microscopy images were taken using the Inspect S50 microscope (FEI Company, Hillsboro, Oregon, USA). The images were taken at ×1000 magnification, with an accelerating voltage of 10 kV and a working distance of 11 mm. A gold layer of approximately 4 nm was applied to the sample.

Time-domain THz spectroscopy was performed by measuring the complex sample transmittance using a custom-made spectrometer based on Ti:sapphire femtosecond laser with an Optistat (Oxford instruments) cryostat. The sample was prepared as a powder in a cuvette (thickness 0.2 mm). Reference measurements with an empty cuvette, which enable a reliable determination of the transmittance, were performed systematically at each temperature value.

A Bruker Equinox 55 FT-IR spectrometer coupled to a Hyperion 2000 microscope was used to measure transmission spectra under temperature and pressure. The spectra were recorded in a CsI matrix (c=1:500 under temperature and 1:100 under pressure) in the mid-infrared region (650-4000 cm^{-1}). The spectral resolution was 2 cm^{-1}. Tungsten lamps were used as the radiation source, and liquid nitrogen-cooled MCT detectors (500-7000 cm^{-1}) were used. For the temperature measurements (from 5 to 300 K) we used CF 2102 cryostat (Oxford instruments) with temperature stability of 0.01 K and a cooling rate of 0.1 $K \cdot min^{-1}$ controlled by Oxford Inst. ITC 503 thermoregulator and PKR 251/26001 flow meter. For the pressure measurements a Merrill-Bassett high-pressure chamber was employed with a stainless-steel gasket as a seal between the diamonds (IIac diamonds, culet size 0.8 mm, embedded in BeCu anvil rings). The pressure inside the chamber increased mechanically when three screws were turned. Cesium iodide (CsI) was used as a medium to transmit the hydrostatic pressure from the anvil pistons to the sample (up to 7 GPa).[2] For each subsequent pressure, the air spectrum was used as a reference. The composite chamber (which contained air at the sample location) was used to record the diamond set's absorption spectrum, which was then cut off from the spectra recorded in this way.

Raman spectra under temperature were recorded using a LabRAM HR 800 UV HORIBA Jobin Yvon spectrometer with a liquid nitrogen-cooled CCD detector. The Raman spectrometer is calibrated using Si as a calibration template after each start-up of the device and after the laser has warmed up (at least once a day). The spectrum was recorded using He–Ne laser excitation (l = 632.8 nm). The spectral resolution was better than 2 cm^{-1}. The laser power at the sample was less than 1 mW. Raman spectra of OrtImi crystal were recorded in three spectral regions, 20–200, 780–915, and 1040–1240 cm^{-1}, using a 20× objective.

Raman scattering under pressure was recorded in a back-scattering geometry using an RM-1000 Renishaw Raman microscope equipped with a double Bragg filter, allowing very good stray light

rejection below 5 cm^{-1}. The Raman spectra were collected using the 632.8 nm line of an He–Ne laser at a power of about 25 mW (~2.5–5 mW on the sample). A Merrill-Bassett diamond anvil was used to record Raman spectra as a function of pressure. Hydrostatic pressure was transferred from the anvil piston to the sample using cesium iodide (CsI) as the medium and a stainless-steel gasket. Type IIac diamonds (0.7 mm culet size) were embedded in BeCu anvil rings.

Ruby crystals were used to determine the value of the pressure applied. The luminescence spectrum of ruby powder in the range of 680-720 nm, which was located in the diamond chamber together with the test sample, was recorded. The ruby spectra were fitted with a Gaussian function in the Fittyk program and the positions of the ν_1 and ν_2 bands were read.[3] Using methods proposed by Dewaele et al.[4], the pressure in the diamond chamber was calculated.

The experimental spectra were deconvoluted in Fityk 0.9.8.5.[5] First, the background was cut off from the set of spectra in the same way, regardless of temperature or pressure. Then, the harmonic-shaped bands were fitted using the Lorentzian function, while the anharmonic bands were fitted using the SplitPearson7 model. Based on these fitted functions, parameters analyzed in the study were extracted, such as position, intensity and half-width.

Quantum chemistry calculations were performed using the Gaussian 09 software package.[6] Potential energy surface (PES) calculations were performed for helix models using the ωB97x-D functional[7,8] with 6-311G (d,p) Pople style basis set.[9] The helical arrangements (1, 2, and 3) chosen for the calculations were based on X-ray structural data, as shown in Figure S3. The model shown in Figure S12 was used to carry out normal modes calculations for the optimized systems. Theoretical computations were carried out at the DFT/ωB97x-D/6-311G++(d,p) level of theory. A description of the change in the O···O distance can be found in Description 3 of the Supplementary Information. Optimization of the system to the energy minimum was conducted for each step of the O···O distance change (range 2.33–3.03 Å, step 0.05 Å), as indicated by the absence of negative frequencies in the theoretical spectra. PES calculations for the systems shown in Figure S11 were performed at the DFT/ωB97x-D/6-311G++(d,p) level of theory. The optimization and calculation of the normal vibrations for ring systems (see Fig. 5) were performed at the level of DFT/ωB97x-D/6-311G++(d,p) theory. The A-ring arrangement is obtained from the optimization of the helical arrangement (a slice of the helix). The B-ring arrangement is optimized from a ring (planar, see fig. 5b) arrangement (blue). A scaling factor of 0.99 was used for the calculated Raman spectra in the range of 400–1300 cm^{-1}, which was calculated from the slope of the dependence of the calculated band positions on their corresponding positions in the experimental spectrum (see Fig. S32).

GaussSum software was used to obtain theoretical IR and Raman spectra.[10] The following parameters were used to obtain the theoretical infrared and Raman spectra: a spectral range of 0–4000 cm^{-1}; 4000 numbers of points in the spectrum; a half-width of 10 cm^{-1}; an excitation line of 633 nm; and a temperature of 293.15 K.

Theoretical calculations based on the Quantum Theory of Atoms in Molecules (QTAiM)[11] were performed to determine the critical points of hydrogen bonds in model systems. The calculations were carried out using AIMAll software.[12] The energies of hydrogen bonds were calculated using the Espinosa relationship: $E_{HB} = 1/2\ V_{BCP}$.[13]

Structural optimizations and molecular dynamics (MD) simulations were carried out using the Atomic Simulation Environment (ASE).[14] The interactions between atoms were described with the foundation (universal) MACE-MH-1[15,16] machine-learning interatomic potential using the omat_pbe head and including D3 dispersion correction.[17,18]

Structural optimizations were performed by relaxing both atomic positions and lattice parameters until forces were below 0.005 eV/Å. MD simulations were conducted in the NPT ensemble. Each simulation began with a 20 ps equilibration using the Berendsen thermostat and barostat, followed by a 40 ps production run employing isotropic Martyna–Tobias–Klein (MTK)[19] NPT dynamics, chosen to reflect the isotropic pressure conditions of the experiment. In the MTK simulations, thermostat and barostat

time constants were set to 100 and 1000 MD timesteps, respectively. Simulations were carried out at pressures ranging from 0 to 20 GPa in 1 GPa increments at a constant temperature of 300 K, with each production run initiated from the Berendsen equilibrated structure obtained at the corresponding pressure.

The scripts used for structural optimization and MD simulations are publicly available on GitHub.[20] These scripts were generated using the uMLIP-Interactive[21] interface to streamline and allow easily reproducible calculations.

The IR response was obtained from the MD simulations by computing the frequency-dependent dielectric permittivity from polarization fluctuations within the linear response theory. The MD trajectory was generated with a time step of 4 fs, providing access to the relevant vibrational frequencies, and a total length of 40 ps, yielding sufficient frequency resolution for comparison with the experiment. Effective atomic charges were derived from ab initio calculations performed using the Gaussian software package for a 54-atom helical structural motif of OrtImi. These charges were kept fixed during the analysis and assigned to the atomic positions along the MD trajectory. The time-dependent polarization P(t) of the simulation cell containing 432 atoms was calculated at each time step according to the classical expression

$$\mathbf{P}(t) = \frac{1}{V}\sum_{i} q_i \boldsymbol{r}_i(t),$$

where q_i and $\mathbf{r}_i(t)$ are the effective charge and position of atom i at time t, respectively, and V is the simulation cell volume at a given pressure. Then, the main components of the frequency-dependent dielectric loss function (imaginary part of the dielectric permittivity) were obtained from the polarization autocorrelation function using the fluctuation–dissipation theorem. The effective permittivity was calculated as the average of three main components and compared to the experimental IR spectra.

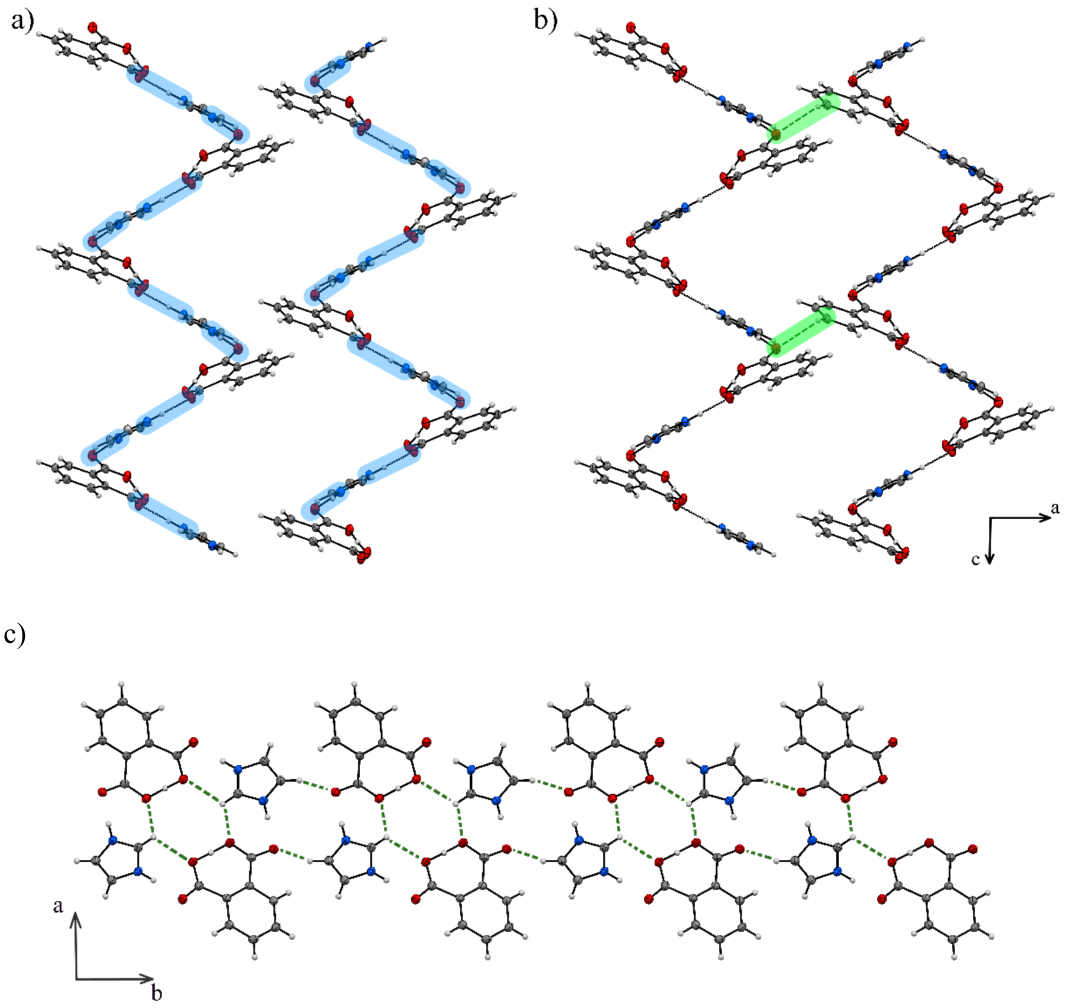

Figure S1. Projection to the *ac* plane of the molecular packing in the crystal of imidazolium hydrogen *o*-phthalate showing ions linked by N1b$^+$–H1b···O8a$^-$ and N2b$^+$–H2b···O11a$^-$ hydrogen bonds to form the helix motif along the *c*-axis (a) and C2b–H2b···O11a connecting adjacent helices (b); The projection of molecules in OrtImi linked by C–H···O hydrogen bonds into layers parallel to the *ab* plane (c).

Description S1. OrtImi crystallizes in the orthorhombic system, space group *Fdd*2, lattice parameters: a=47.177(2), b=22.9080(9), c=3.78426(16) Å, V=4089.7(3) Å^3, Z=16, T=130.0(1) K, CCDC (Cambridge Crystallographic Data Centre) number: 1847597.[1] Ions are connected by N1b$^+$–H1b···O8a$^-$ ($d_{D\cdots A}$=2.74 Å, E_{HB}= -9.55 kcal·mol^{-1}) and N2b$^+$–H2b···O11a$^-$ ($d_{D\cdots A}$=2.75 Å, E_{HB}= -9.38 kcal·mol^{-1}) (Fig. 1, S1a, and Tab. S1) and can be described by $C_2^2(12)\vec{a}\overleftarrow{b}$ chain motif of hydrogen bonds (HBs).[22] The helix chains grow along the *c*-axis. In anions, short intramolecular O···H···O$^-$ HBs occur ($d_{D\cdots A}$=2.38 Å, E_{HB}= -83.88 kcal·mol^{-1}, S(7) intramolecular HB motif). Anions and cations are also connected by weak C1b–H1b···O12a ($d_{D\cdots A}$=3.01 Å, E_{HB}= -1.86 kcal·mol^{-1}) and C2b–H2b···O11a ($d_{D\cdots A}$=3.25 Å) HBs (Fig. 1a, S1b, and S2). The C2b–H2b···O11a HBs connect adjacent helices. All HBs stabilize the position of ions in the crystal structure.

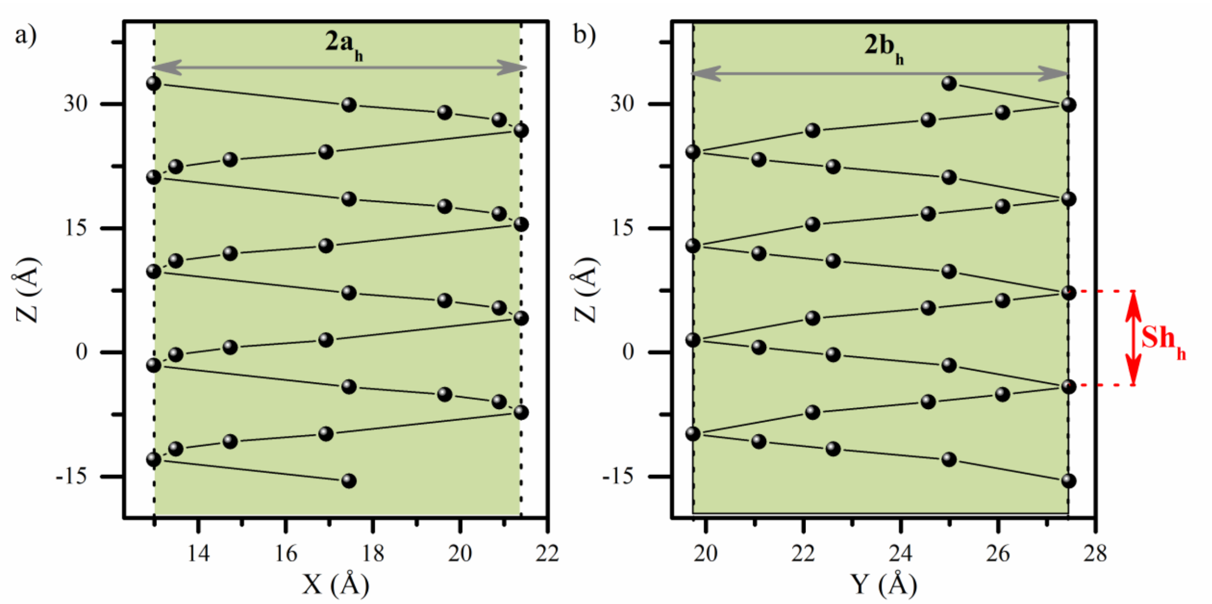

Figure S2. Projection on the xz (a) and yz (b) planes of the helix formed in the imidazolium hydrogen *o*-phthalate crystal structure at 130 K under atmospheric pressure. Meaning of symbols: Sh_h – helix pitch, a_h – semi-major axis, and b_h – semi-minor axis. The black dots show where oxygen and nitrogen are located in the helical HB network; *a*//x, *b*//y, *c*//z.

Table S1. Calculated QTAiM parameters (in atomic unit) corresponding to the H···O bond critical point (BCPs), the electron density at BCP, ρ_{BCP}; Laplacian of electron density at BCP, Δ_{BCP}; total electron energy density at BCP, H_{BCP}, and its components: kinetic electron energy density, G_{BCP} and potential electron energy density, V_{BCP}; hydrogen bond energy, E_{HB} (kcal·mol^{-1}).

	Type of HB	ρ_{BCP}	Δ_{BCP}	G_{BCP}	V_{BCP}	H_{BCP}	E_{HB} (kcal·mol^{-1})
HB 1	N^+–H···O^-	0.0357	0.1209	0.0303	-0.0304	-0.0001	-9.53
HB 2	O···H···O^-	0.1743	-0.2874	0.0979	-0.2677	-0.1698	-83.98
HB 3	N^+–H···O^-	0.0349	0.1229	0.0303	-0.0298	0.0005	-9.36
HB 4	N^+–H···O^-	0.0353	0.1255	0.0309	-0.0304	0.0005	-9.55
HB 5	O···H···O^-	0.1735	-0.2783	0.0989	-0.2674	-0.1685	-83.88
HB 6	N^+–H···O^-	0.0349	0.1232	0.0303	-0.0299	0.0005	-9.38
HB 7	N^+–H···O^-	0.0356	0.1235	0.0307	-0.3055	0.0002	-9.58
HB 8	O···H···O^-	0.1772	-0.3154	0.0957	-0.2703	-0.1746	-84.79

HB – hydrogen bond; Figures 1 (in the main paper) and S3a show the HB 1-8 bonds.

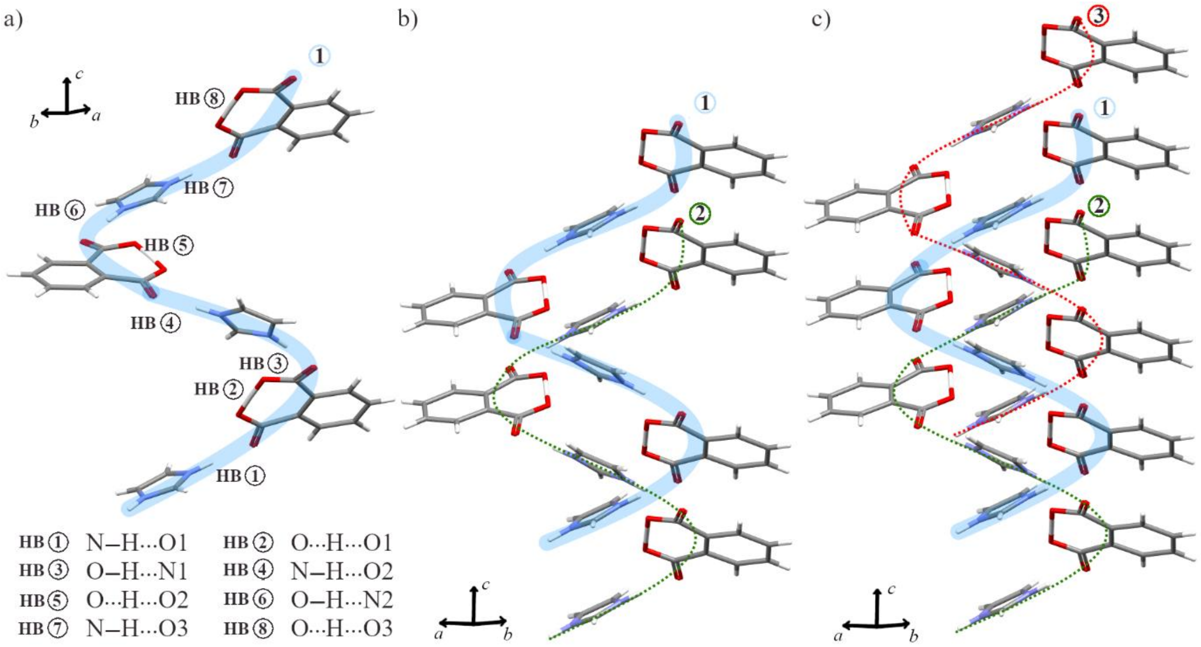

Figure S3. Arrangement of ions in the OrtImi crystallographic structure, for which Potential Energy Surface (PES) calculations were performed using DFT methods: one (a), two (b), and three (c) helices. All three helicoids are formed along a single axis in the [001] direction and are left-handed.

Description S2: To check the influence of the environment on the shape and size of the energy barrier, calculations for helix 1 were performed on three systems consisting of one (Fig. S3a), two (Fig. S3b), and three (Fig. S3c) helices. For helical systems, the energy barriers that a proton would have to overcome when transferring from the HB donor to its acceptor were calculated (see Fig. S4 and Tab. S2). The calculations revealed that the most significant differences in potentials were observed for intermolecular HBs outside the helical system (HB1 and HB7). The calculations suggest that weak π···π interactions between helices slightly deform the potential barrier, deepening the energy minimum (see Figure S4). For this reason, the energies and shapes of the potential wells presented in the paper are appropriate for the following bonds: HB5 (O···H···O^{-}) and HB4 and HB6 (N^{+}–H···O^{-}) (see Fig. 1b or S3a for the description of the bonds).

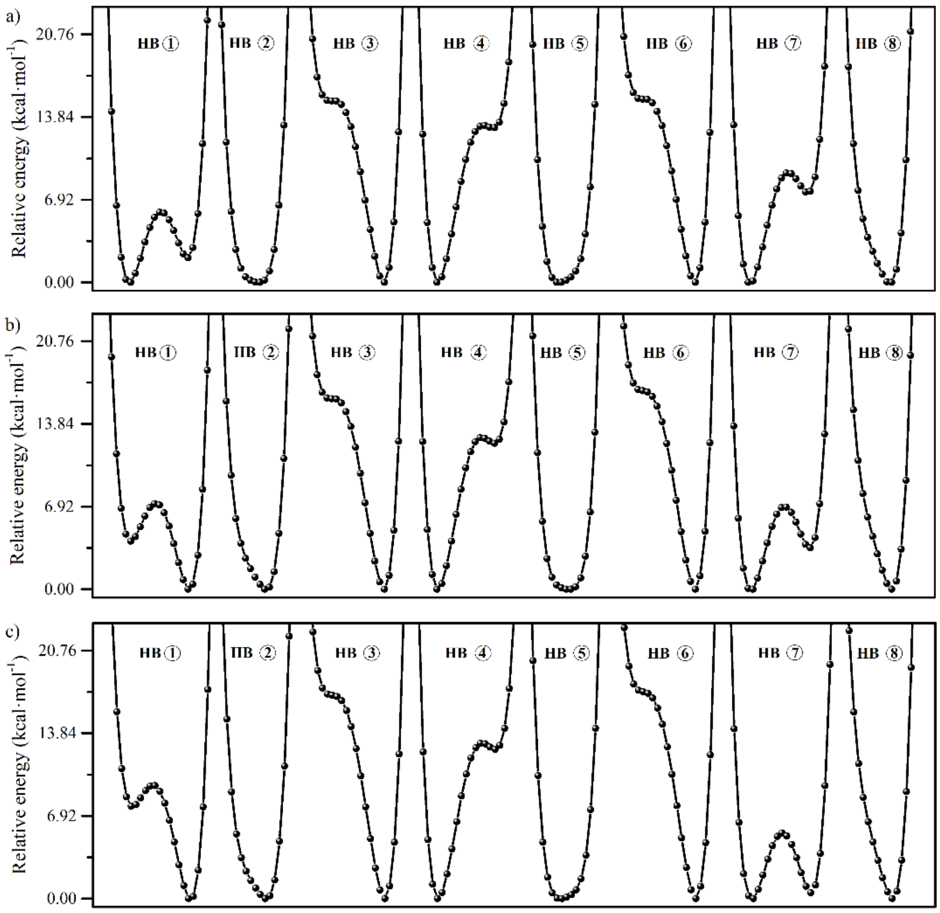

Figure S4. The energy barriers that a proton must overcome in the transferring process in hydrogen bonds N^+–H···O^- and O···H···O^- are shown for three systems consisting of (a) one (cf. Fig. S3a), (b) two (cf. Fig. S3b), and (c) three (cf. Fig. S3c) helices. See Figure S3a for the description of the bonds.

Table S2. The total energies of the helical systems (helix 1, helices 2, and helices 3, see Fig. S3) and the relative barrier energies for N^{+}–H···O^{-} hydrogen bonds.

		Total Energy (kcal·mol^{-1})		
		Helix 1	Helices 2	Helices 3
HB1	SP1	-1572702.4	-3145431.78	-4718188.6
	TS	-1572696.5	-3145428.6	-4718185.2
	SP2	-1572700.3	-3145435.8	-4718196.3
HB2	SP1	-1572693	-3145422.6	-4718178.6
HB3	SP1	-1572685.1	-3145412.5	-4718167.4
	TS	-1572685.1	-3145412.5	-4718167.4
	SP2	-1572700.3	-3145428.5	-4718184.4
HB4	SP1	-1572685.1	-3145429.1	-4718185.2
	TS	-1572685.1	-3145416.4	-4718172.2
	SP2	-1572700.3	-3145416.9	-4718172.7
HB5	SP1	-1572693	-3145421.3	-4718177.5
HB6	SP1	-1572684.9	-3145411.8	-4718167.1
	TS	-1572684.9	-3145411.8	-4718167.1
	SP2	-1572700.3	-3145428.4	-4718184.3
HB7	SP1	-1572701.4	-3145430.2	-4718186.8
	TS	-1572692.3	-3145423.4	-4718181.4
	SP2	-1572693.8	-3145426.7	-4718186.3
HB8	SP1	-1572694.9	-3145424.8	-4718181.2
		Relative Energy (kcal·mol^{-1})		
HB1	TS-SP1	5.9	3.2	3.4
	TS-SP2	3.8	7.2	11.1
HB3	TS-SP1	0	0	0
	TS-SP2	15.2	16	17
HB4	TS-SP1	15.2	12.7	13
	TS-SP2	0	0.5	0.5
HB6	TS-SP1	0	0	0
	TS-SP2	15.4	16.6	17.2
HB7	TS-SP1	9.1	6.8	5.4
	TS-SP2	1.5	3.3	4.9

Legend: TS – Transition state, SP – Stationary point.

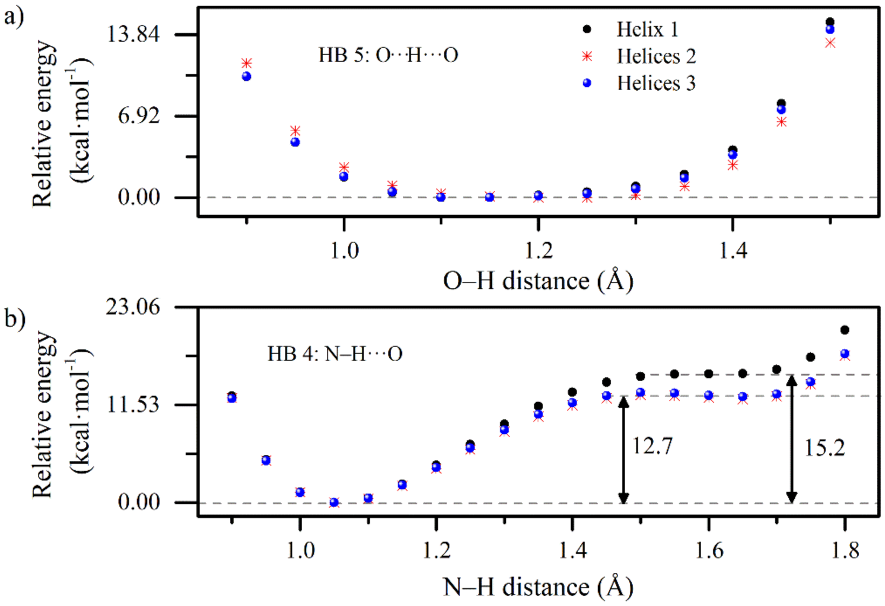

Figure S5. Potential energy surface (PES) calculations were performed to study the tunnelling motion of a proton between donor and acceptor atoms in O··H··O (a) and N–H···O (b). The barrier was calculated for systems consisting of one (black), two (red), and three (blue) helices. See Figure S3a for a description of bonds.

Description S3: Infrared absorbance, THz, and non-polarized Raman spectra were measured to analyze the vibrational properties of OrtImi (see Fig. S6); The polarized Raman spectra are shown in Figs. S7 and S8. The measured spectra were compared to the theoretical calculations made using DFT methods to assign the observed bands (Fig. S9-S10). The molecular arrangement for which these calculations were performed is shown in Fig. S11. The measured spectra can be divided into several regions corresponding to some characteristic excitations: the valence band information is encoded in the spectral range from 3000 to 3500 cm^{-1},[1,23] the fingerprint spectrum corresponds to the 500–1800 cm^{-1} range, and, finally, the lattice vibrations (phonons) oscillate in 5–500 cm^{-1} range.[24,25]

The assignments of important vibrational bands observed by FTIR and Raman scattering are shown in Table S2. The band at 2982 cm^{-1} is related to νO–H+γO··H··O and the band at 1924 cm^{-1} to νO–H (only FTIR spectrum and see Supplementary Description S3 and Fig. S12). Two intense bands at 1058 and 992 cm^{-1}, observed in the infrared spectrum are associated with out-of-plane deformational vibrations γ(O··H··O). These bands are broad and characterized by an asymmetric shape. This is related to the proton in O–H···O, which never oscillates individually, but rather cooperatively couples and resonates with electrons to facilitate tautomerization of the molecule.[26,27]

In the IR spectrum bands at 3099, 3075, and 3068 cm^{-1} are related to stretching vibration of νN–H. These bands appear in the Raman spectra for $z(xy)\bar{z}$, $z(yy)\bar{z}$, $z(xx)\bar{z}$ and $z(yx)\bar{z}$ (Figure S8). The reason for this is that N$^+$–H···O$^-$ HBs in the helical structure are arranged along the *x* and *y* directions (parallel to the *a* and *b* parameters of the elementary cell). The 896, 853, and 794 cm^{-1} bands are associated with γCNH and γCNN deformation, respectively (Fig. S6).

In the 5–200 cm^{-1} range, we observe bands associated with lattice oscillations (see Fig. S13, Table S2).[28] Within this range, the bands observed are associated with longitudinal and transverse optical phonons.[24,25] In Fig. S6 we divide this range into three narrower intervals: 5–70 cm^{-1} (Z1), 70–130 cm^{-1} (Z2), and 130–290 cm^{-1} (Z3). Z_1, Z_2, and Z_3 are associated with out-of-plane deformation phonons γNHO+γOHO, in-plane deformation phonons δNHO, and stretching phonons νNHO+νOHO, respectively (see Table S2).[25,27,29] The theoretical analysis of these bands is presented in description S4. We observe 11 phonon modes labelled ν_1-ν_{11} (see Fig. S6a). Most bands are associated with both N$^+$–H···O$^-$ and O··H··O$^-$ HBs. The reason for this is that the O··H··O$^-$ HB structures are intramolecular bonds that do not link to other structures in the crystal lattice. However, they do affect the dynamics of the N$^+$–H···O$^-$ HBs. In the polarization spectrum (Figure S7a), these bands are most intense in the *y*-

direction (parallel to *b*), where both the $N^+–H\cdots O^-$ and the $O\cdots H\cdots O^-$ HBs make a significant contribution.

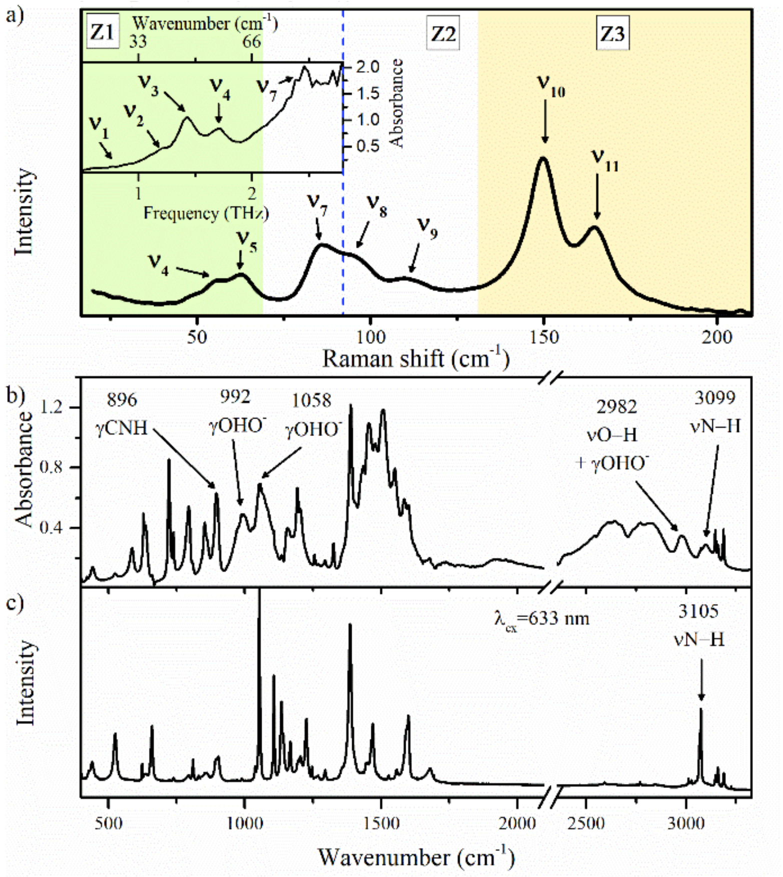

Figure S6. Raman (16-210 cm^{-1}) and THz (10-98 cm^{-1}) (insert in panel) (a), FTIR (b), and Raman (c) from 400 to 3330 cm^{-1} spectra of OrtImi. The THz spectrum was obtained with an OrtImi powder in an infrasil cuvette (thickness equals 0.2 mm) and IR absorption was measured with OrImi in KBr matrix (*c*=1:1000).

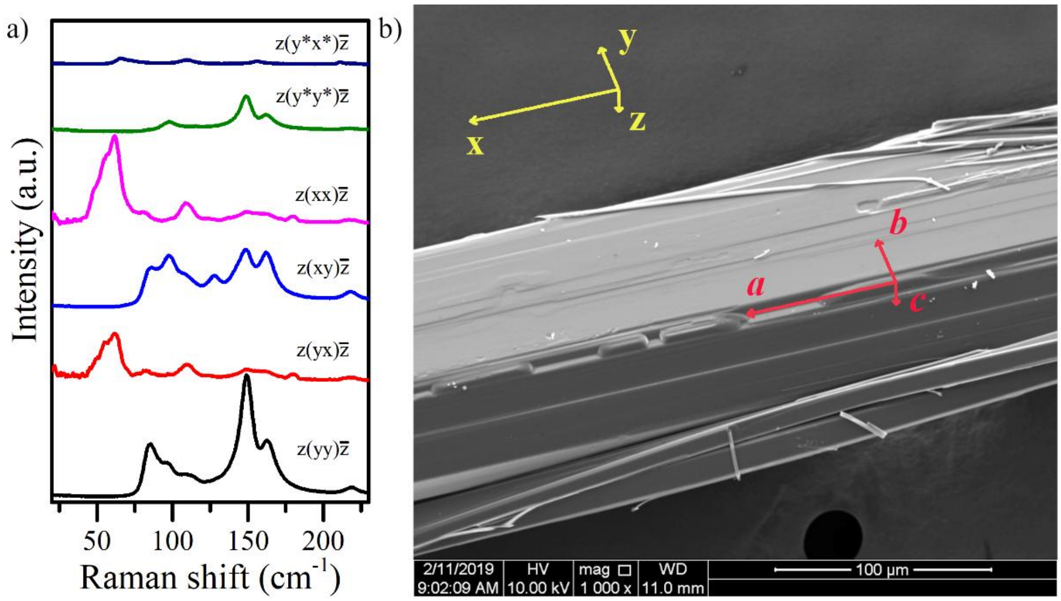

Figure S7. Raman spectra of OrtImi in the range of 20–220 cm^{-1}, depending on polarization (a); SEM image with unit cell axes (marked in red) and the chosen laboratory coordinate system (yellow) (b). Note: $a \parallel x, b \parallel y, c \parallel z$.

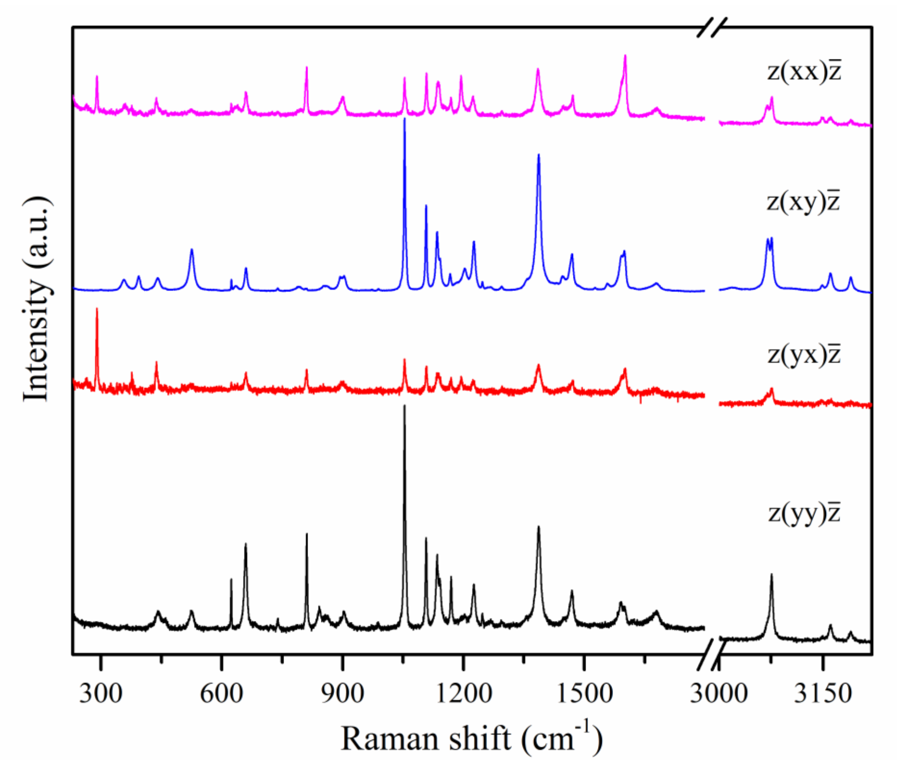

Figure S8. Polarization-resolved Raman spectra of OrtImi in the range from 250 to 3200 cm^{-1}.

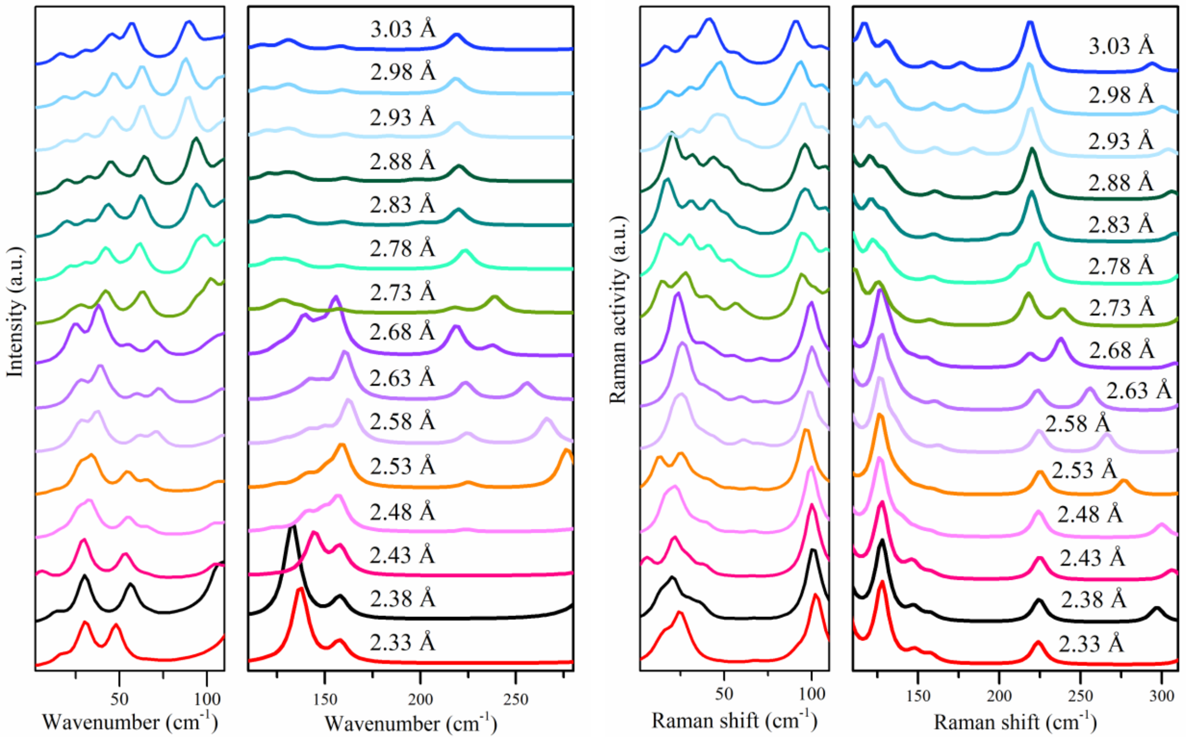

Figure S9. Calculated dependence of the IR (on the left) and Raman (on the right) spectra on the length of the O–H···O⁻ HB ($d_{O\cdots O}$) in the range 0-280 cm^{-1}. The considered system is depicted in Figure S11. DFT method: ωB97x-D/6-311++G(d,p).

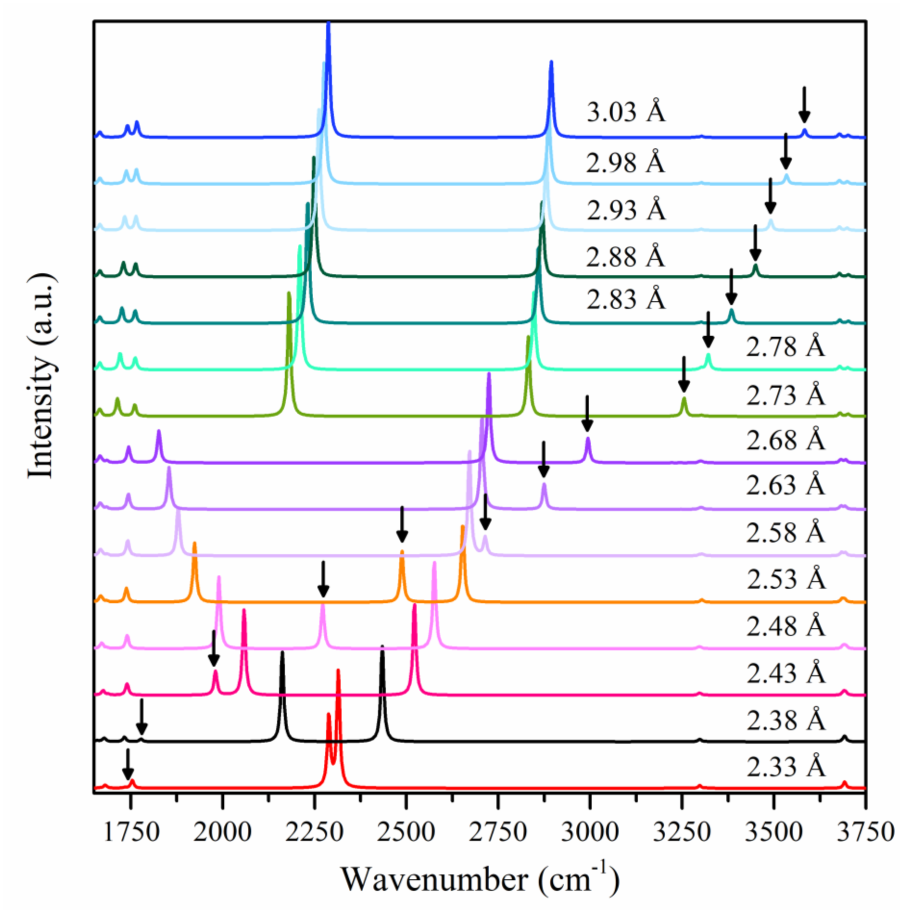

Figure S10. Calculated dependence of the IR spectra on the length of the O–H···O⁻ HB ($d_{O\cdots O}$) in the range 1725-3750 cm^{-1}. The considered system is depicted in Figure S11. DFT method: ωB97x-D/6-311++G(d,p). The arrows indicate the band associated with the stretching vibration of the O–H bonds in the O–H···O⁻ HB.

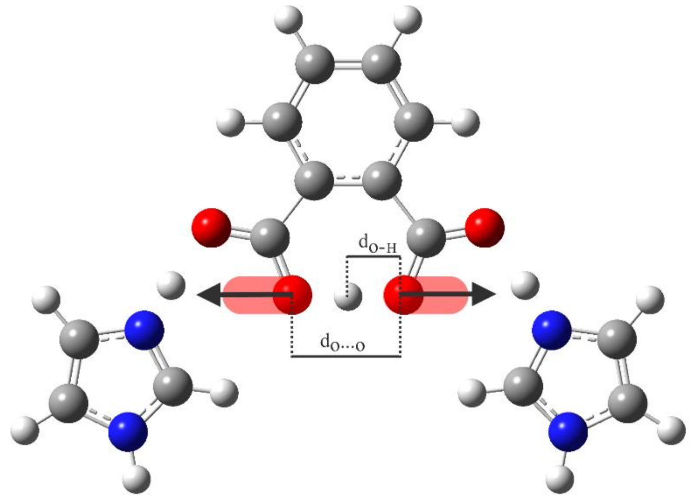

Figure S11. The ion arrangement in OrtImi for which optimization calculations were performed depends on the length of the O–H···O⁻ HB. Based on this, we calculated the following: IR and Raman spectra. Labels: $d_{O\cdots O}$ – O–H···O⁻ hydrogen bond length and $d_{O\text{–}H}$ – oxygen–hydrogen bond length.

Table S3. The assignment of the selected vibrational bands observed in FTIR and Raman spectra of OrtImi. The bands were assigned according to the literature and DFT calculations.

	$\nu_{FT\text{-}IR}$ (cm^{-1})[a]	ν_{Raman} (cm^{-1})[a]	assignment	Literature
1	3188m	3189w	νC–H	30
2	3161w	3160w	νC–H	30
3	3148m	3148w	νC–H	30
4	3099m	3105w	νN–H	31
5	3075w	3075s	νN–H	28,32
6	3068vw	3070s	νN–H	28,32
7	2982m		νO–H+γO···H···O	27,28,31
8	1924w		ν(O–H)	33
9	1600m	1601m	νC=C (C=C bond within the benzene and imidazole ring)	1
10	1585m		$\nu(COO^-)_{as}$	1
11	1549m		ν(C–N)	34
12	1506s		ν(C–COO)	23,35
13	1469s	1469w	νC=C (C=C bond within the benzene and imidazole ring)	1
14	1455s		ring mode + δ(CNH)	24,36
15	1434m		νC–N	34
16	1388vs		$\nu(COO^-)_{sym}$	1
17		1387s	ν(C–OH)	27,37
18		1225w	νC–N	34
19	1203m	1203vvw	Benzene δC–H	1
20	1156w	1167m	δ(CNH)+δ(CNC)	1
		1145m	δ(CNH)+δ(CNC)	1
21	1135vvw	1134m	δ(CNH)+δ(CNC)	1
22		1107m	Benzene δC–H	1
23	1058s		$\gamma(OHO^-)_{as}$	1,38
24		1053vs	νC–C	1
25	992m		$\gamma(OHO^-)_{as}$	38
26	896s		γ(CNH)	1
27	853m		γ(CNH)	1
28	808vw	810w	γ(CCC)	1
29	794s		γ(CCN)+δ(COO)	1
30	770s		Hydrogen atoms of ortho-substituted aromatic rings	1
31	721s		δ(C–OH)	1
32		659w	δ(NCC)	36
33	636m		δ(CNC)	36
34	586w		δ(CCH)	1
35	523vw	524m	δ(CNH)	34
36	441w	440w	δ(OHO)	27
37		180	$\nu(O \cdots H \cdots O^-)+\nu(N^+–H \cdots O^-)$	24,32,33,39,40
38		ν_{11} 164.9	$\nu(N^+–H \cdots O^-)$	25,27,29
39		ν_{10} 149.7	$\nu(O \cdots H \cdots O^-)+\nu(N^+–H \cdots O^-)$	25,27,29
40		ν_{p9} 109.7	$\delta(N^+–H \cdots O^-)$	24,41
41		ν_8 94.6	$\gamma(N^+–H \cdots O^-)$	24,41
42	ν_7 80.5 (2.44 THz)	ν_7 85.9	$\gamma(N^+–H \cdots O^-)$	24,41
43	ν_6 74.9 (2.27 THz)		$\gamma(N^+–H \cdots O^-)$	24,41
44		ν_5 62.6	$\gamma(O \cdots H \cdots O^-)$	24,25,41
45	ν_4 55.8 (1.69 THz)	ν_4 55.6	$\gamma(N^+–H \cdots O^-)+\gamma(O \cdots H \cdots O^-)$	24,25,41
46	ν_3 47.2 (1.43 THz)		$\gamma(N^+–H \cdots O^-)+\gamma(O \cdots H \cdots O^-)$	24,25,41
47	ν_2 39.6 (1.20 THz)		$\gamma(N^+–H \cdots O^-)+\gamma(O \cdots H \cdots O^-)$	24,25,41
48	ν_1 31.4 (0.95 THz)		weak interactions inside the helix	25

[a] Intensities: vs, very strong; s, strong; m, medium; w, weak; vw, very weak.

Description S4: An intense band associated with O–H bond stretching is typically observed in the infrared spectrum in the 3000–3500 cm^{-1} range.[33] However, the position of this band changes when the O–H bond is part of an O···H···O HB. A strong red shift of this band indicates the presence of O···H···O HB coupling.[27] To determine the position of the ν_{O-H} band in the infrared spectrum, calculations were performed to establish its position as a function of the donor–acceptor length in the O···H···O⁻ HB ($d_{O\cdots O}$; see Fig. S10). The calculations were performed for a variable $d_{O\cdots O}$ length from 2.33 Å up to 3.03 Å with a step of 0.05 Å (Fig. S11 shows the system for which the calculations were performed). As the donor-acceptor distance decreases, the band red shifts significantly and the O–H bond elongates (see Fig. S10 and Fig. S12). In the range of 2.33–2.43 Å, the position of the νO–H band is between 1735 and 1980 cm^{-1} (Fig. S10). For strong hydrogen bonds, this band is also characterized by a very low intensity (see Figs S10 and S12). To determine the position of the band at room temperature for OrtImi, we estimated it from the difference between the positions of the bands at 2982 cm^{-1} (νO–H+γOHO⁻) and 1058 cm^{-1} (γ(OHO⁻)). The band related to νO–H is located at 1924 cm^{-1} and exhibits a very low intensity (Fig. S12). This result is consistent with calculations performed on strong O–H···O HBs and with the literature.[27,33]

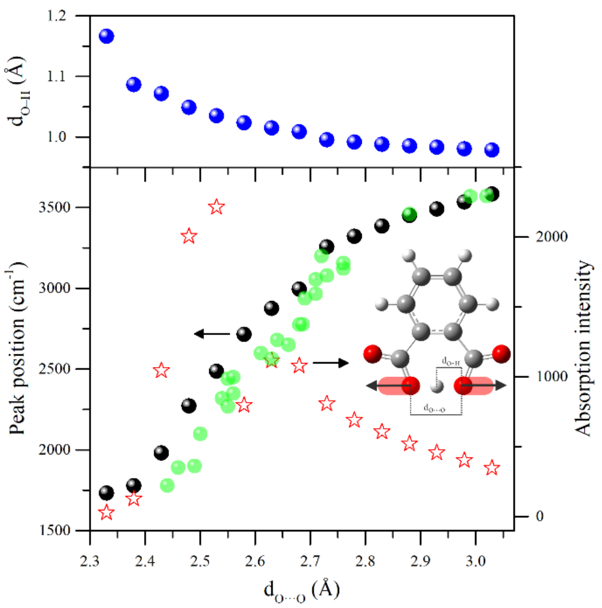

Figure S12. The dependence of the d_{O-H} bond length (upper panel) and of the position and absorption intensity of the νO–H band (lower panel) as a function of the O···O distance in the O–H···O⁻ HB calculated by DFT/ωB97x-D/6-311++G(d,p). The inset in the bottom panel shows the anion structure and indicates the direction of increasing O···O distance. Green point (lower panel) position of the experimental νO–H band as a function of the O···O distance in the O–H···O HB from the Ref.[42]

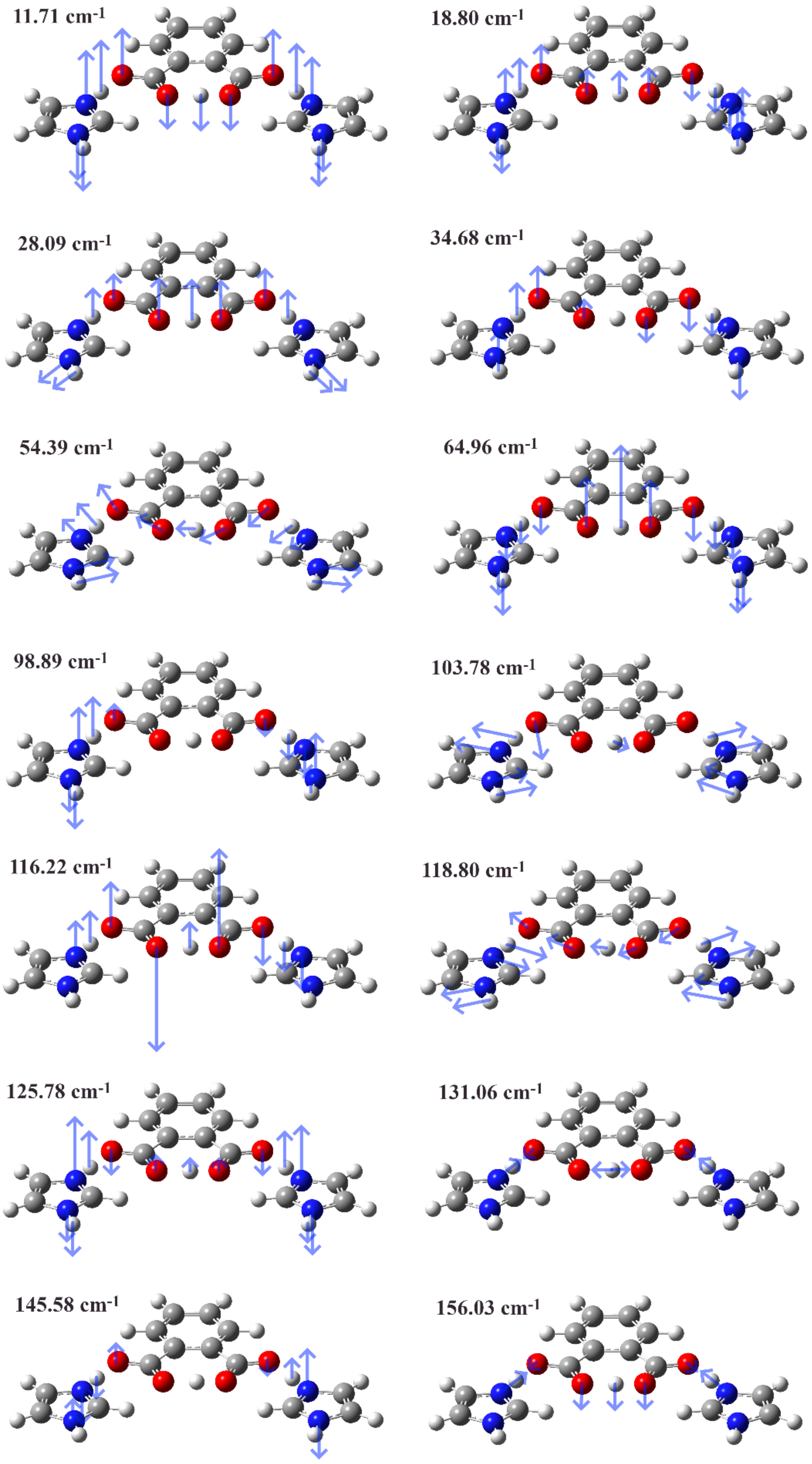

Figure S13. The Eigenvector Displacement (blue arrows) for the vibrational modes observed in the theoretical IR and Raman spectra of OrtImi is shown for the 0–200 cm^{-1} range.

Description S5: The DFT/ωB97x-D/6-311++G(d,p) method was used to calculate the Raman and infrared (IR) spectra as a function of the O–H···O⁻ HB length (see Figs. S9-S10; Fig. 11 shows the system for which the calculations were performed). The calculated spectra in the skeletal vibration range 0–200 cm^{-1} were used to interpret the phonon vibrations observed in the experimental Raman and THz spectra. Within this range, 14 modes associated with the collective vibrations of the N–H···O⁻ and O··H··O⁻ HBs in the helical network were observed (see Fig. S13). These differ in direction and intensity, as indicated by the blue arrows in Fig. S13. The bands located at 11.71, 18.80, 28.09, 34.68, and 64.96 cm^{-1} are associated with out-of-plane deformation vibrations of the N–H···O⁻ and O··H··O⁻ HBs in the longitudinal direction of the helical network. The bands at 54.39, 103.78, and 118.80 cm^{-1} are associated with in-plane deformation vibrations of the N–H···O⁻ and O··H··O⁻ HB networks (perpendicular to the helical network). The bands at 131.06, 145.58, and 156.03 cm^{-1} are associated with in-plane stretching of N–H···O⁻ and O··H··O⁻ HBs. The band at 98.89 cm^{-1} is associated with deformation vibrations of N–H···O⁻ hydrogen bridges. The bands at 116.22 and 125.78 cm^{-1} are associated with out-of-plane deformation vibrations of the O··H··O⁻ and N–H···O⁻ HBs networks, respectively. These vibrations force the movement of neighboring HBs.

We observe 12 band components in the experimental THz and Raman spectra (see Table S3). The vibrations at 11.71 and 18.80 cm^{-1} were not observed in the experiment. Although DFT calculations accurately predict the number of bands, they have difficulty representing the intensity ratios accurately. Based on the positions of the bands in the DFT calculations and the literature, phonon vibrations were assigned in OrtImi (see Table S3).

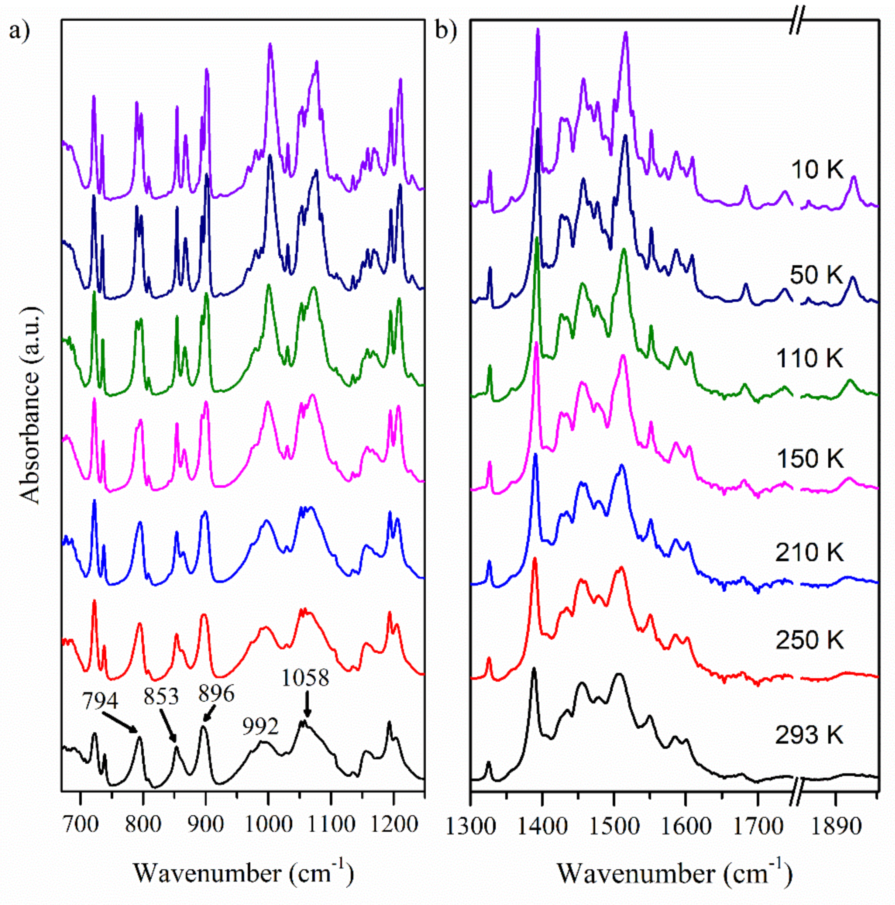

Figure S14. FT-IR spectra of OrtImi, recorded in the KBr matrix (c=1:500) as a function of temperature in two ranges: 670-1250 cm^{-1} (a) and 1300-2000 cm^{-1} (b).

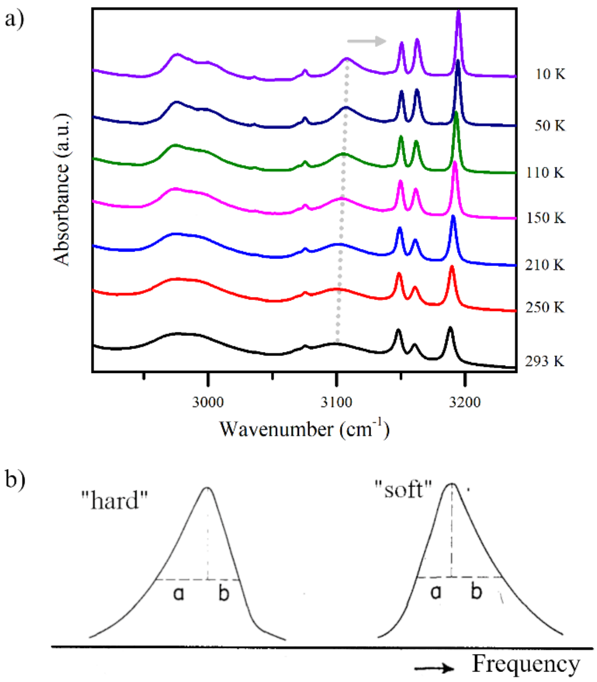

Figure S15. FT-IR spectra of OrtImi in 2910-3340 cm^{-1}, recorded in the KBr matrix as a function of temperature (a). The SplitPearson7 function's parameters a and b are suitable for the "hard" function a>b (on the left) and the "soft" function b>a (on the right) (b).

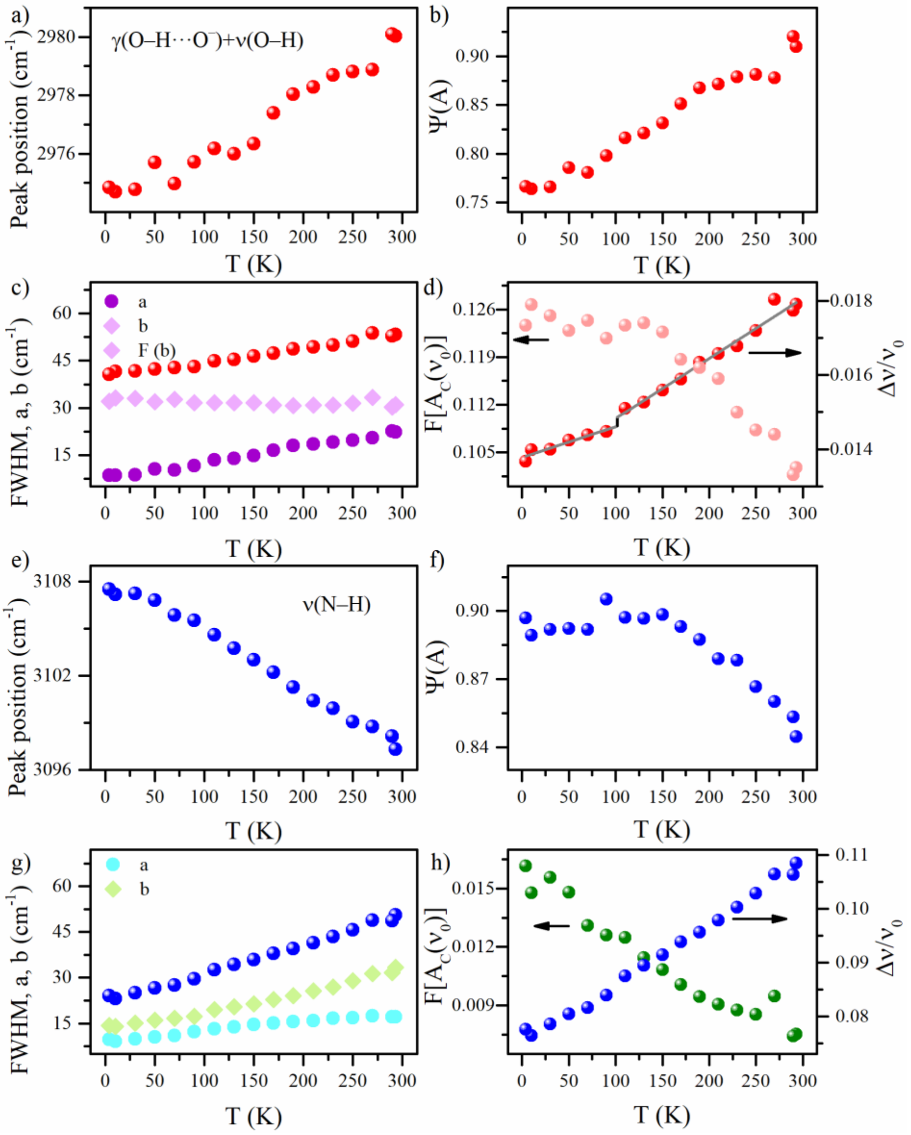

Figure S16. Peak position ν_0 (a, e), anharmonic factor Ψ(A) (b, f), FWHM and the high- and low-frequency *a* and *b* (c, g), and damping parameter $\Delta\nu/\nu_0$ and maximum energy absorption $F[A_C(\nu_0)]$ (d, h) of OrtImi as a function of temperature. Bands connected to γ(O···H···O⁻)+ν(O–H) are depicted in the upper part (a,b,c,d) and those connected to ν(N–H) are shown in the lower part (e,f,g,h).

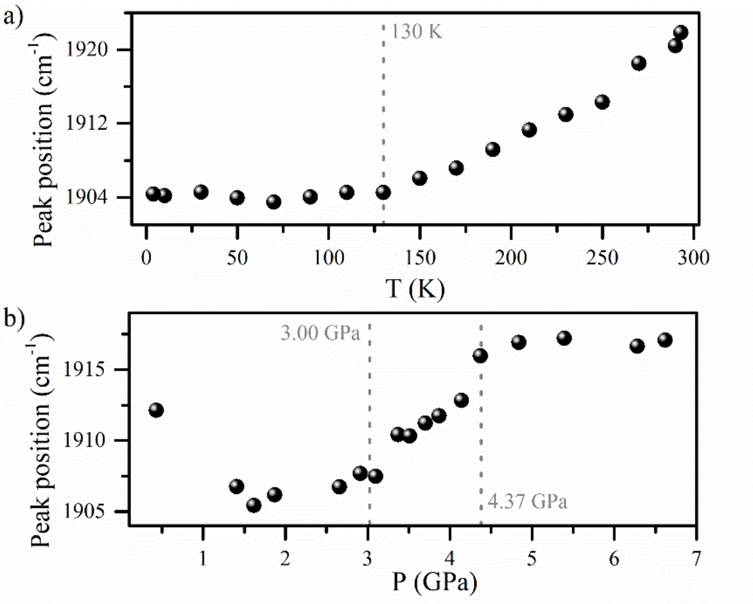

Figure S17. The dependence of the position of the 1924 cm^{-1} band on temperature (a) and pressure (b).

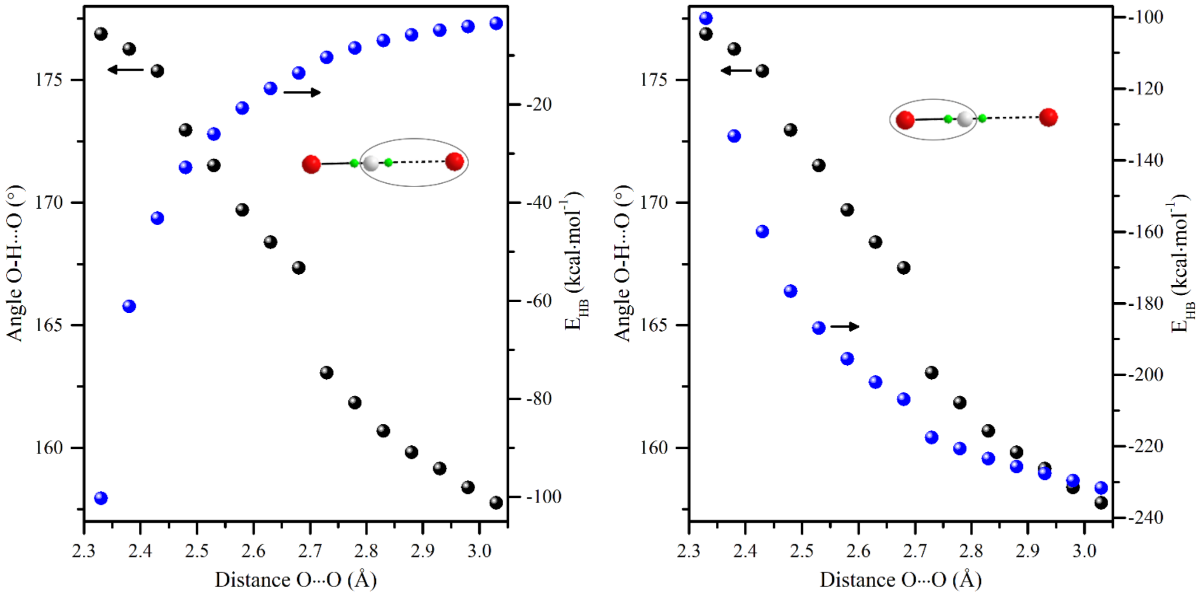

Figure S18. The O–H···O$^-$ hydrogen bond angle (black dots) and hydrogen bond energy (blue) are shown as a function of donor-acceptor distance in the O–H···O$^-$ HB. The energies were calculated based on the BCP (critical point) of the H···O bond (marked with a grey circle, on the left) and of the H–O bond (on the right).

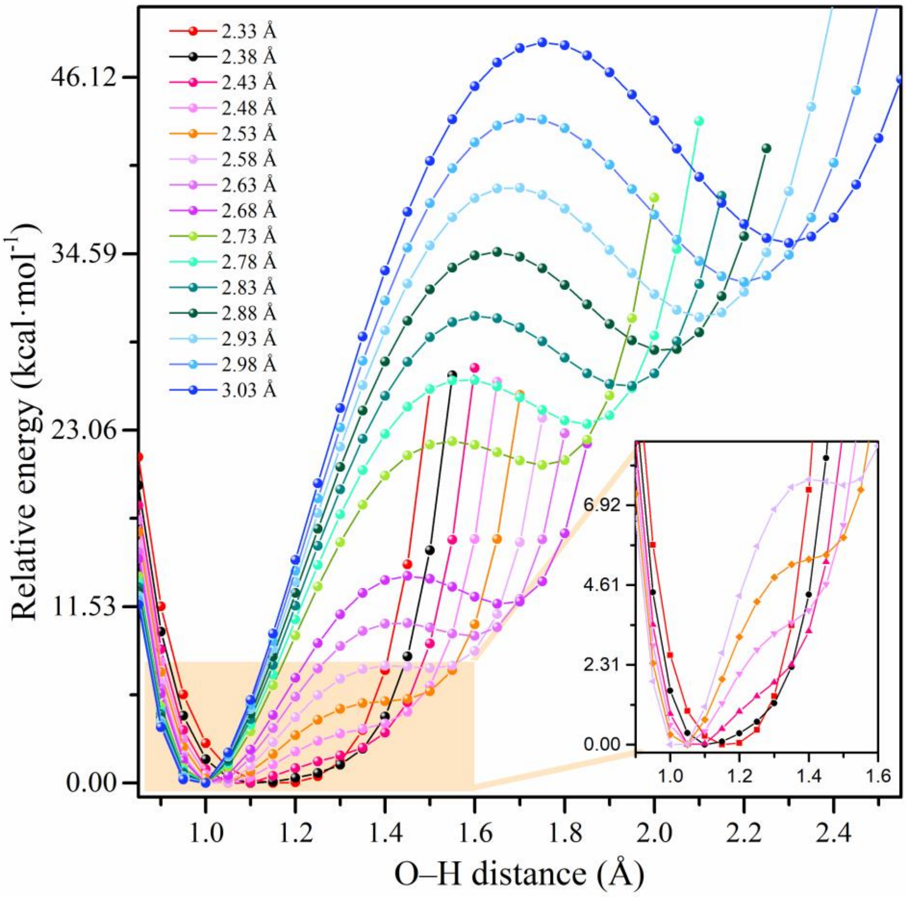

Figure S19. Energy barriers for proton transfer along O–H···O⁻ HBs as a function of donor-acceptor distance. Figure S11 shows the system for which the calculations were performed. DFT method: ωB97x-D/6-311++G(d,p).

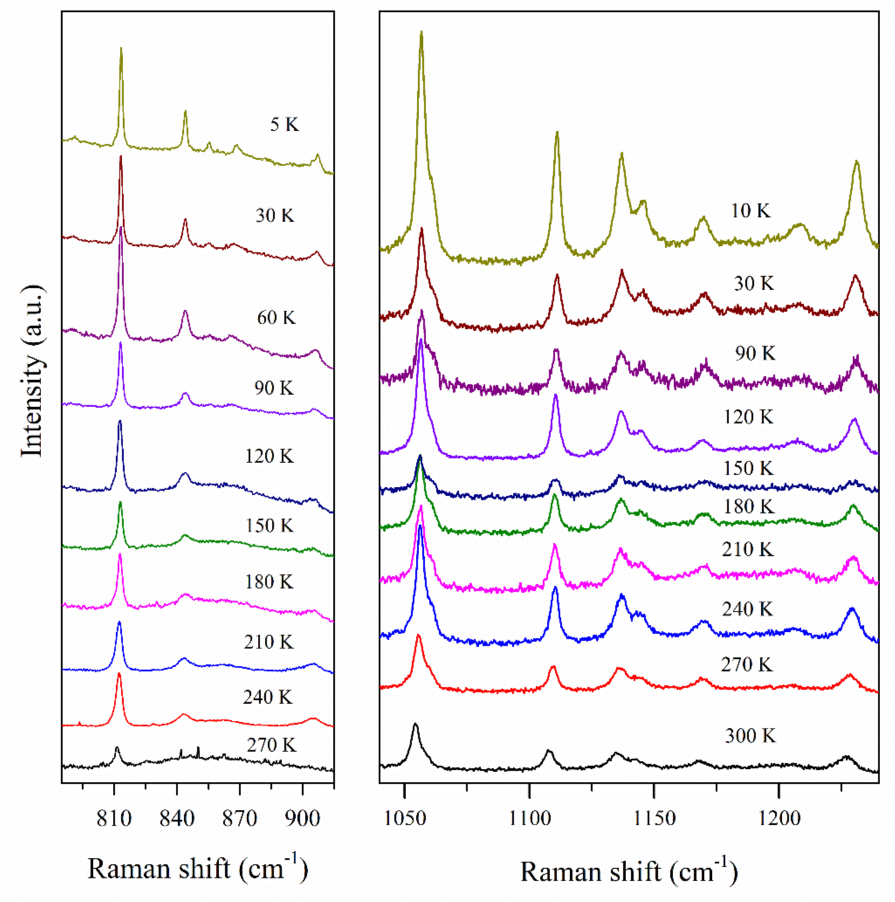

Figure S20. Raman spectra of OrtImi, recorded as a function of temperature in two ranges: 785-915 cm^{-1} (left) and 1040-1240 cm^{-1} (right).

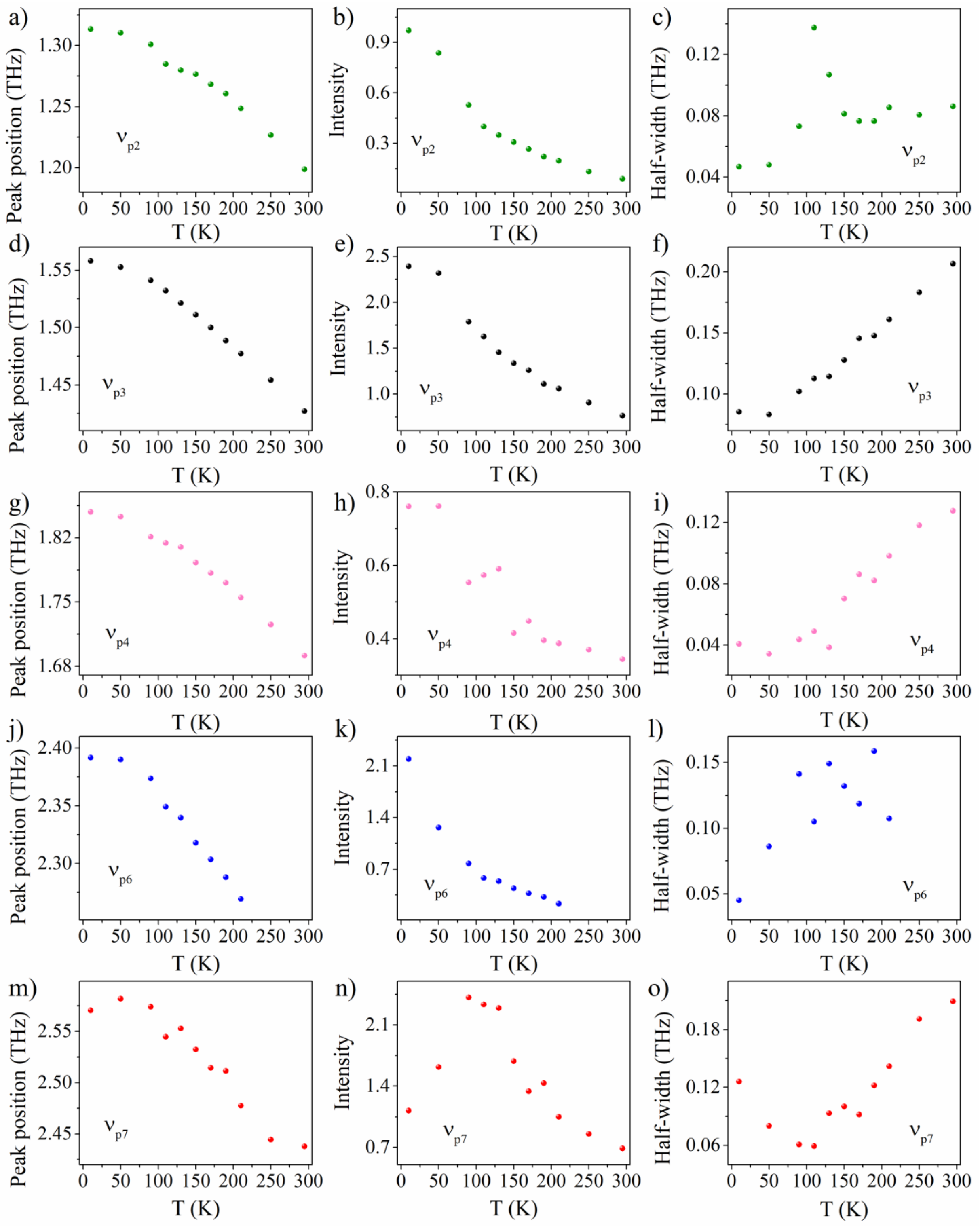

Figure S21. The dependence of the position, intensity, and half-width of the THz OrtImi peaks on temperature for the bands ν_2 (a–c), ν_3 (d–f), ν_4 (g–i), ν_6 (j–l), and ν_7 (m–o) (defined at the Fig. 3a in the main paper).

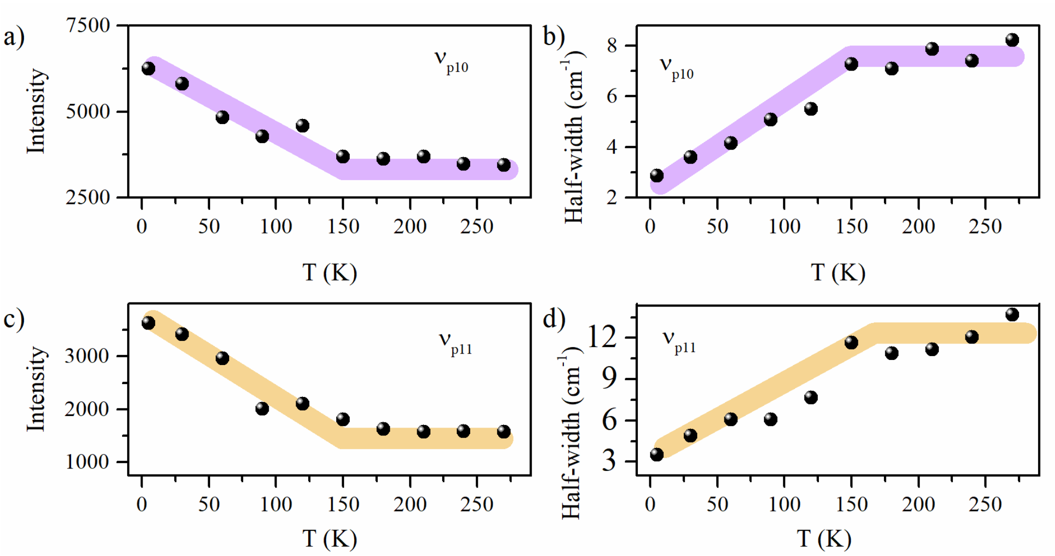

Figure S22. The dependence of the intensity and half-width of the Raman bands in OrtImi on temperature for the bands ν_{10} (a–b) and ν_{11} (c–d).

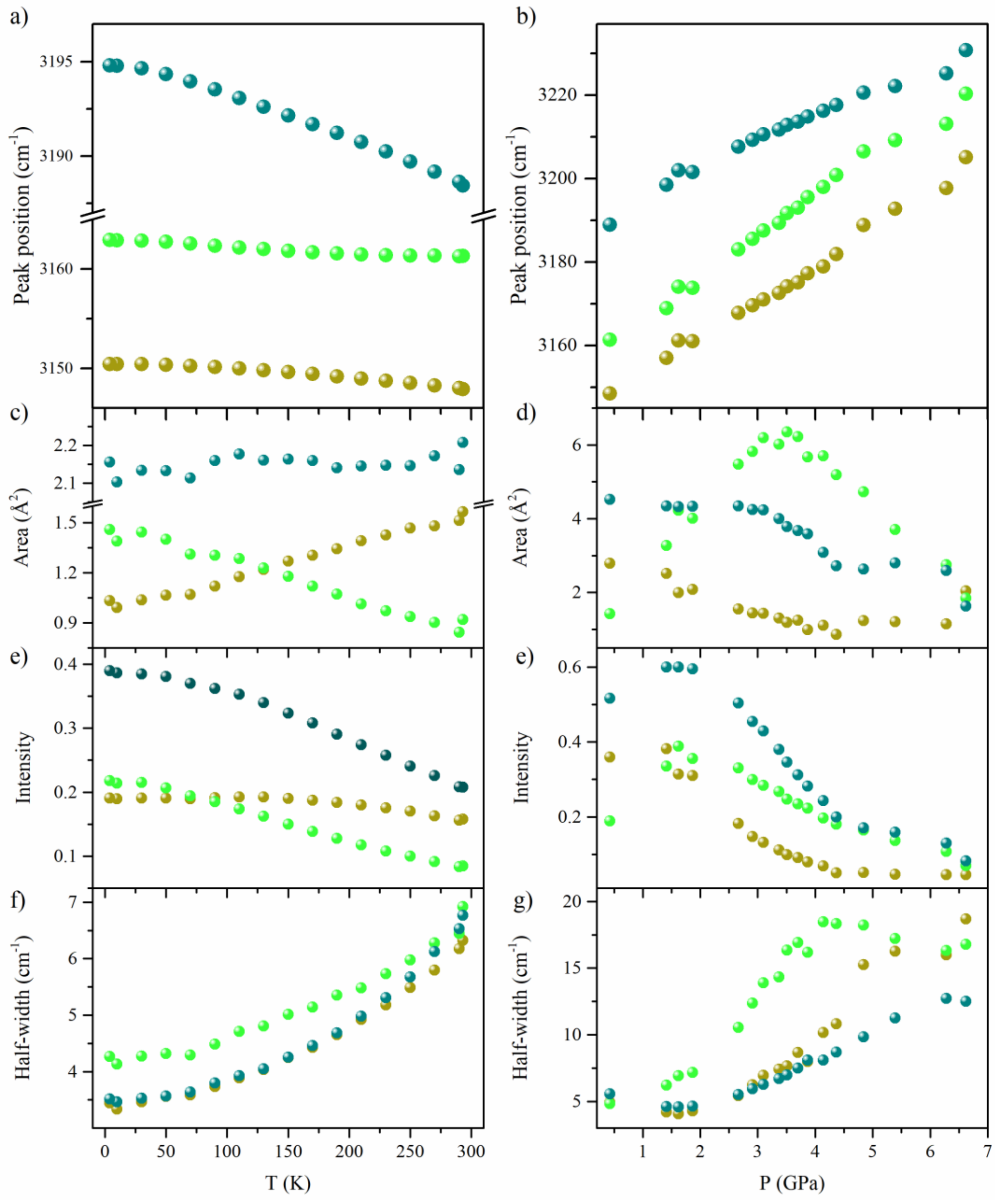

Figure S23. The dependence of the position of the IR νC–H bands in the range 3120–3250 cm^{-1} (a, b), their surface areas (c, d), intensities (e, f), and half-widths (f, g) on temperature and pressure, respectively.

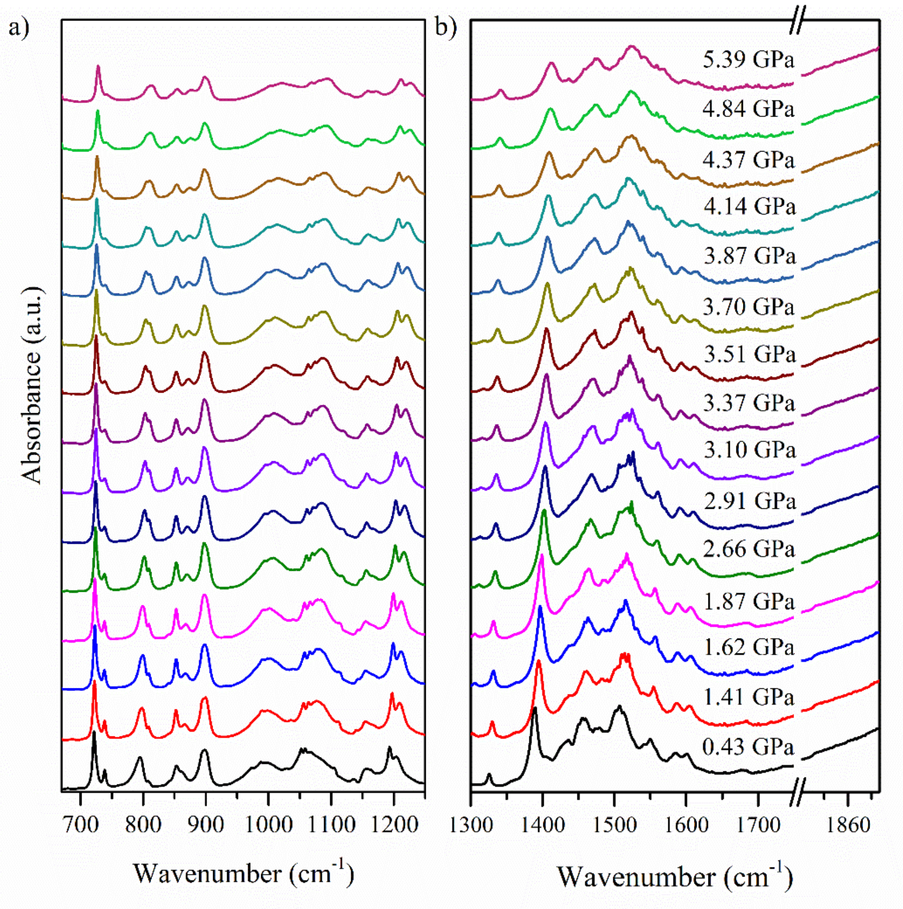

Figure S24. IR spectra of OrtImi, recorded in the CsI matrix (c=1:100) as a function of pressure in two ranges: 670-1250 cm^{-1} (a) and 1300-1900 cm^{-1} (b).

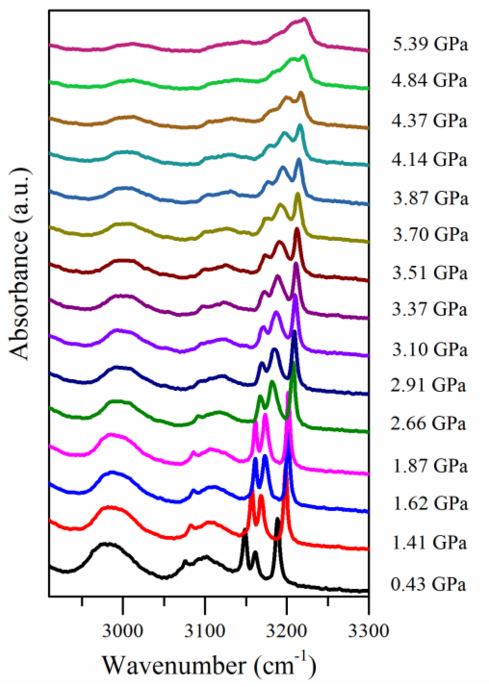

Figure S25. IR spectra of OrtImi, recorded in the CsI matrix (c=1:100) as a function of pressure in 2910-3300 cm^{-1}.

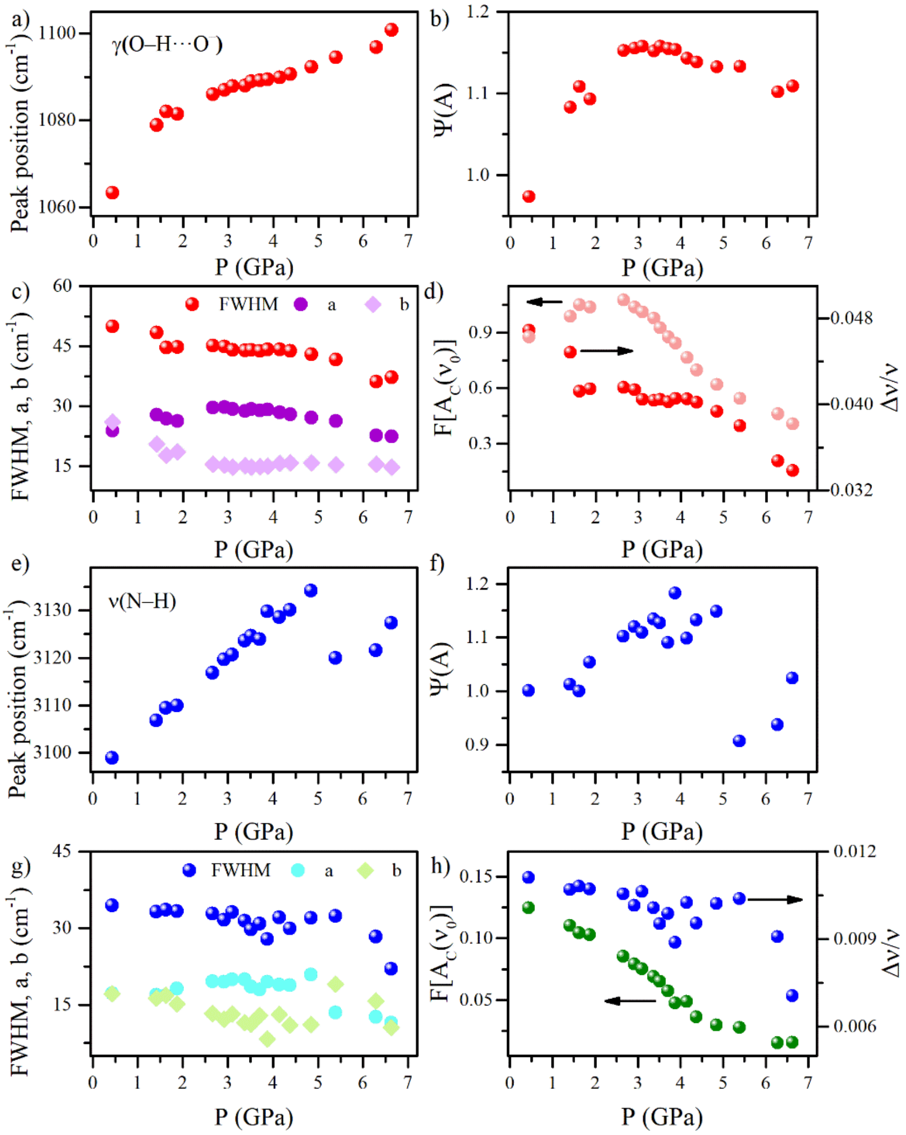

Figure S26. Peak position ν_0 (a, e), anharmonic factor Ψ(A) (b, f), FWHM and the high- and low-frequency HWHMs *a* and *b* (c, g), and damping parameter $\Delta\nu/\nu_0$ and maximum energy absorption $F[A_C(\nu_0)]$ (d, h) of OrtImi as a function of pressure. Bands connected to $\gamma(O{\cdots}H{\cdots}O^-)$ are depicted in the upper part (a,b,c,d) and those connected to ν(N–H) are shown in the lower part (e,f,g,h).

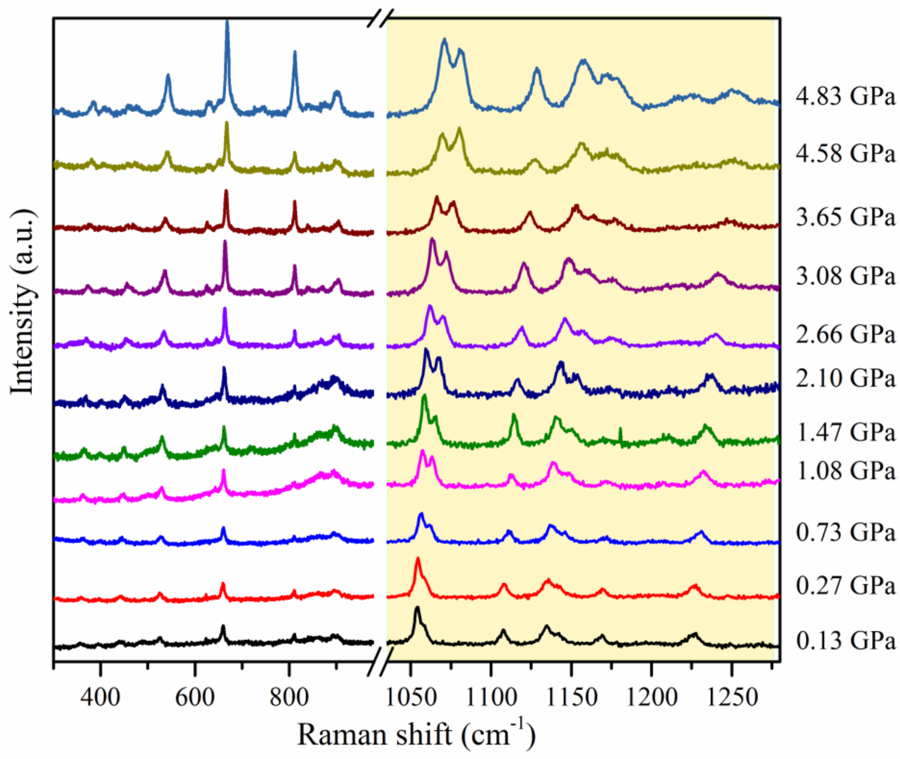

Figure S27. Raman spectra of OrtImi, recorded as a function of pressure in two ranges: 300-980 cm^{-1} (left) and 1035-1280 cm^{-1} (right).

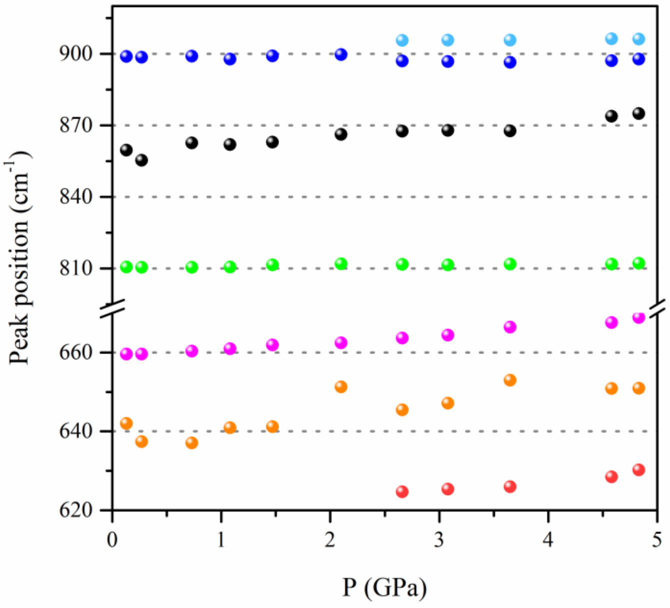

Figure S28. The position of the Raman OrtImi bands in the 620–930 cm^{-1} range depends on pressure.

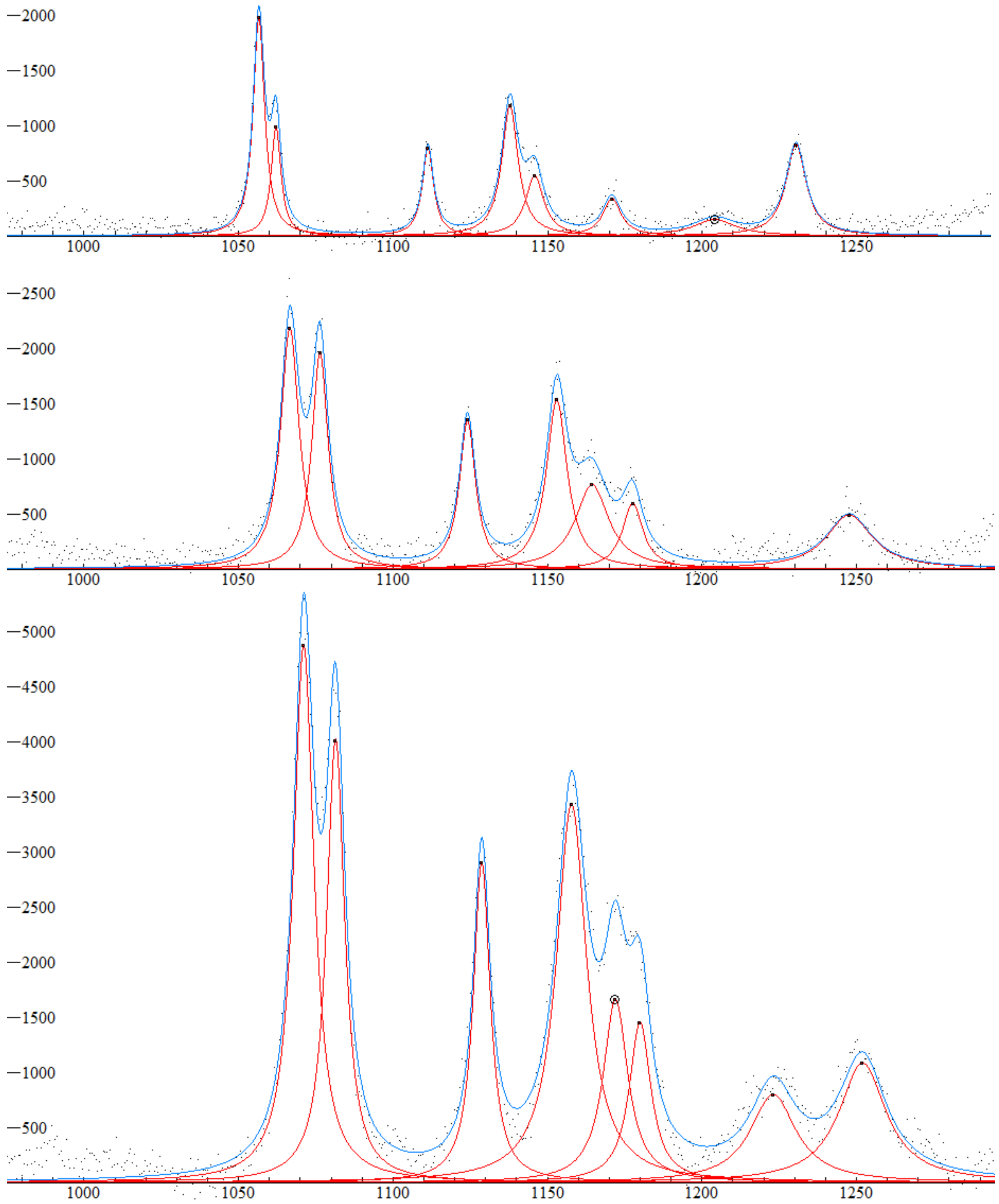

Figure S29. Fits of Raman bands observed in 1000-1300 cm-1 spectral range for OrtImi. Pressures: 0.73 GPa (top), 3.65 GPa (middle) and 4.83 GPa (bottom). These were fitted using the Lorentz function in the Fityk 0.9.8 program.[5]

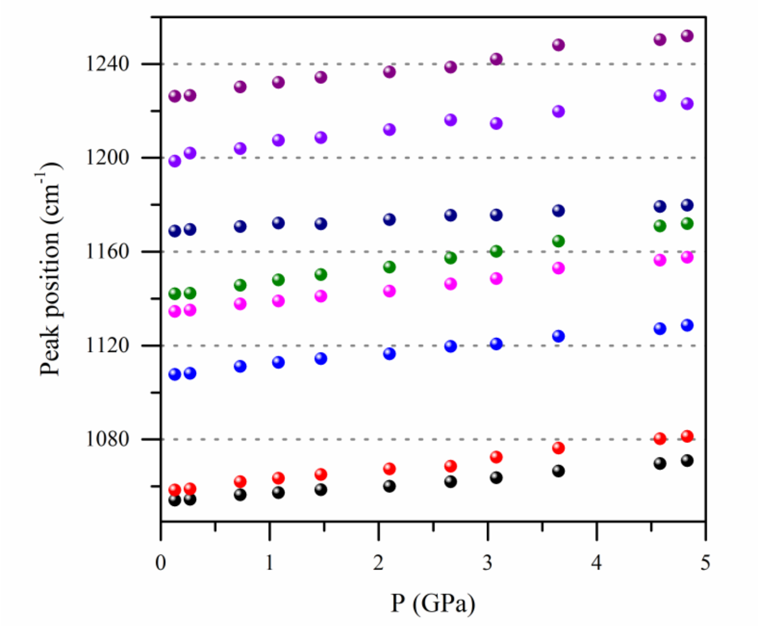

Figure S30. Pressure dependence of the bands in the 1000–1300 cm^{-1} range for OrtImi.

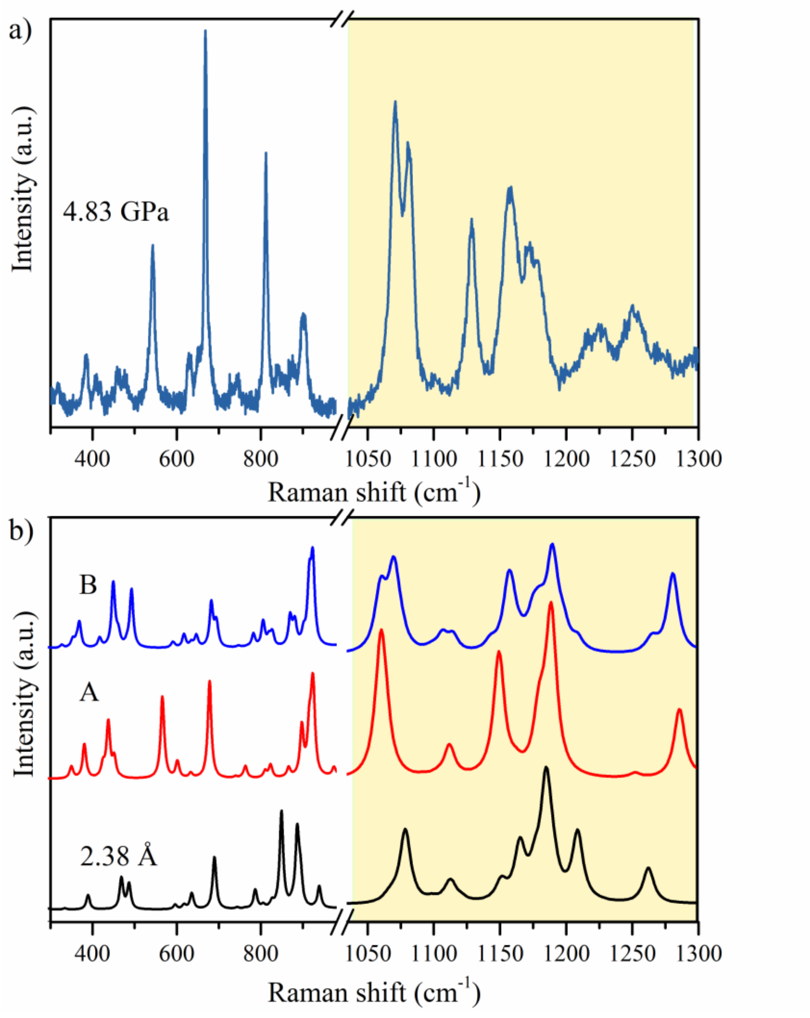

Figure S31. Raman spectrum of OrtImi in the range of 300–1300 cm^{-1}, recorded at a pressure of 4.83 GPa (a). Calculated Raman spectra for systems A and B (see Fig. 5), and for three molecules with O···H···O⁻ hydrogen bonds of length 2.38 Å (Fig. S11). A scaling factor of 0.99 was applied to the calculated spectra (b).

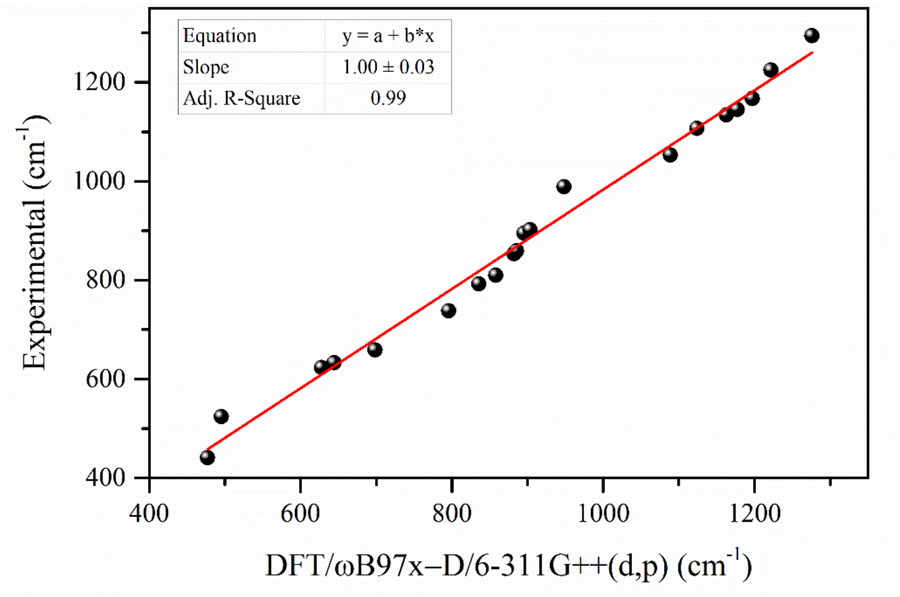

Figure S32. Dependence of calculated and experimental peak positions. The scaling factor for the 400-1300 cm^{-1} range was determined using a linear function fit.

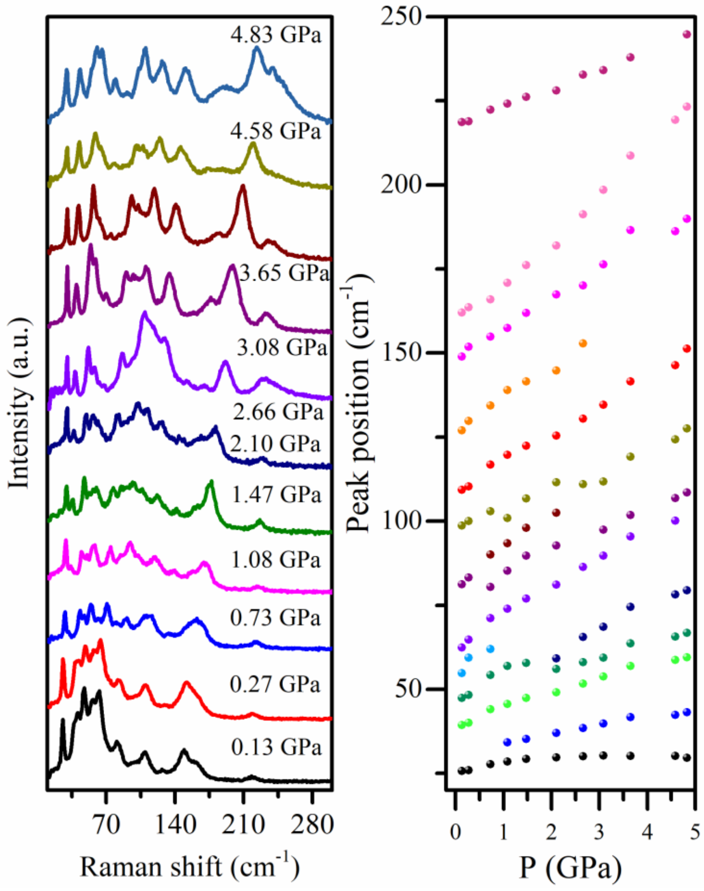

Figure S33. Raman spectrum of OrtImi as a function of pressure in 10-300 cm^{-1} (left) and the dependence of the position of Raman peaks as a function of pressure (right).

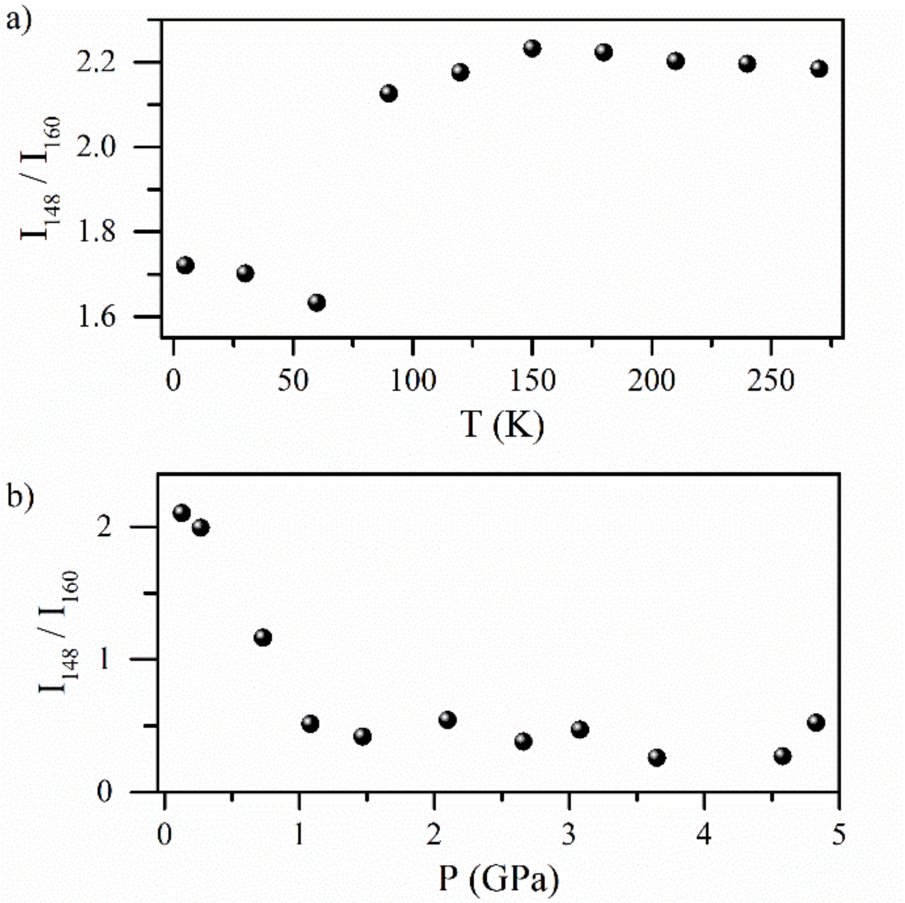

Figure S34. The dependence of the ratio of the intensities of the 148 and 160 cm^{-1} bands on temperature (a) and pressure (b).

Description S6: To simulate the behavior of the helical structure under high pressure, we considered a system of four ions of the crystallographic structure as a fragment of the helix. Optimization of this initially helical system converges to a planar and symmetrical arrangement with N^+–H···O^- HBs forming a ring motif (Fig. 5, system A). If the initial configuration of the fragment is chosen such that the ions are arranged approximately in a ring motif, the optimization shows that a deeper energy minimum corresponds to an approximately flat fragment, which is, however, not completely planar and symmetrical (Fig. 5, system B). A comparison of the experimental and calculated Raman spectra revealed that, at high pressures, the B-system is closer to the experimental results. The reason is that under pressure the helix collapses but is unable to achieve an ideal planar geometry because of the nearest surroundings.

Based on the calculations, the new band observed at high pressures at 625 cm-1 is associated with the deformation of the CNC bonds in the imidazole ring of the system B (Fig. S31). Similar bands occur at 608 cm^{-1} in PyrHem·H_2O in which similar system of ring N^+–H···O^- HBs exists.[43] The spectrum recorded for OrtImi at 4.83 GPa was compared with the calculated spectra for the systems A and B (Fig. S31). For both A and B systems, which correspond to the non-helical configuration of ions, we found that the 1134, 1145, and 1167 cm^{-1} bands present a similar shape to those observed in the experiments, i.e, the 1145 and 1167 cm^{-1} bands overlap while the 1134 cm^{-1} band is detached from them.

Description S7: This potential represents the current state of the art in accuracy for describing materials, molecular systems, and condensed phases, making it a reasonable choice for modeling the pressure-dependent behavior. As an initial validation of this approach, geometry optimization was carried out to assess whether the MACE-MH-1 can reproduce the experimentally observed crystal structure. The optimized structure maintained the original symmetry and overall atomic arrangement of the

experimental structure.[1] Following this validation, MACE-MH-1 was employed to study possible structural changes as a function of increasing pressure. Throughout all simulations, the average temperature remained stable at the target 300 K. The average pressure, however, was systematically lower than the nominal set pressure by approximately 0.5 GPa. This offset persisted even after adjustments to the barostat parameters. This systematic deviation, however, does not affect the qualitative conclusions of the study, only it shifts the explored pressure range to approximately -0.5 to 20.5 GPa.

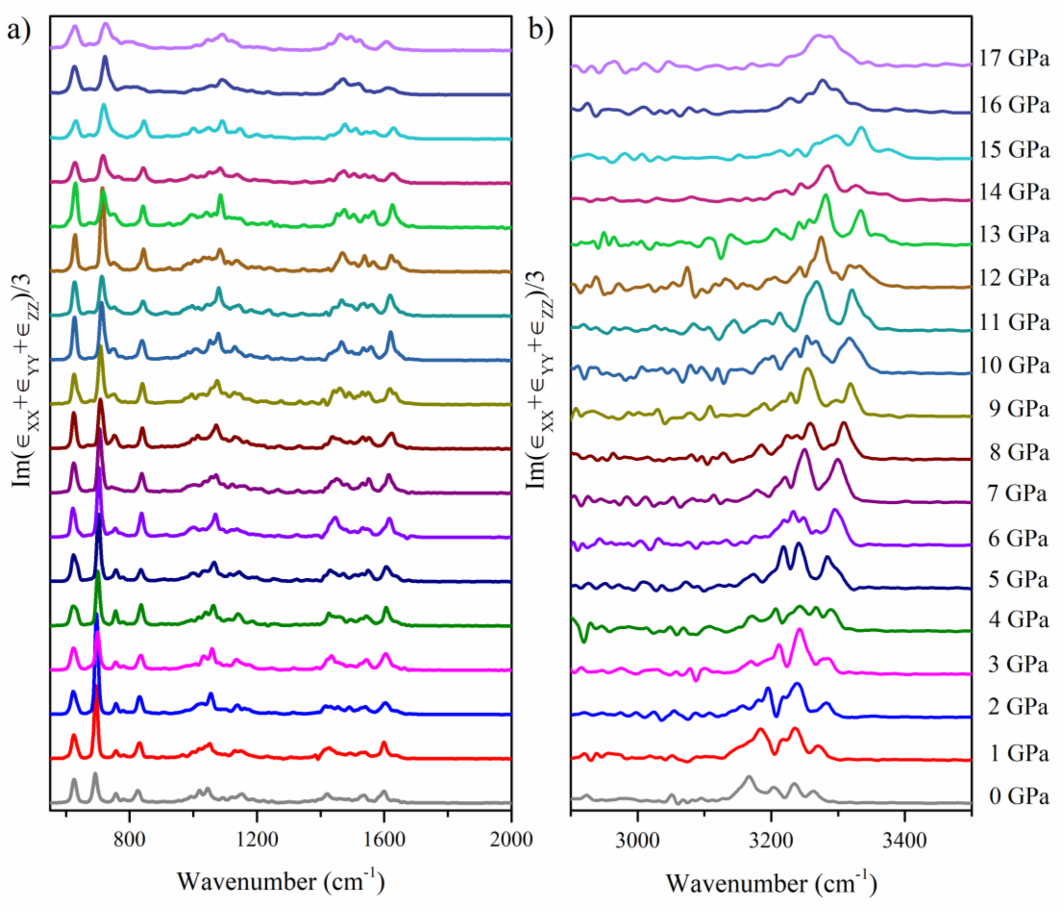

Figure S35. FTIR spectra of OrtImi, calculated as a function of pressure in two ranges: 550-2000 cm^{-1} (a) and 2900-3500 cm^{-1} (b).

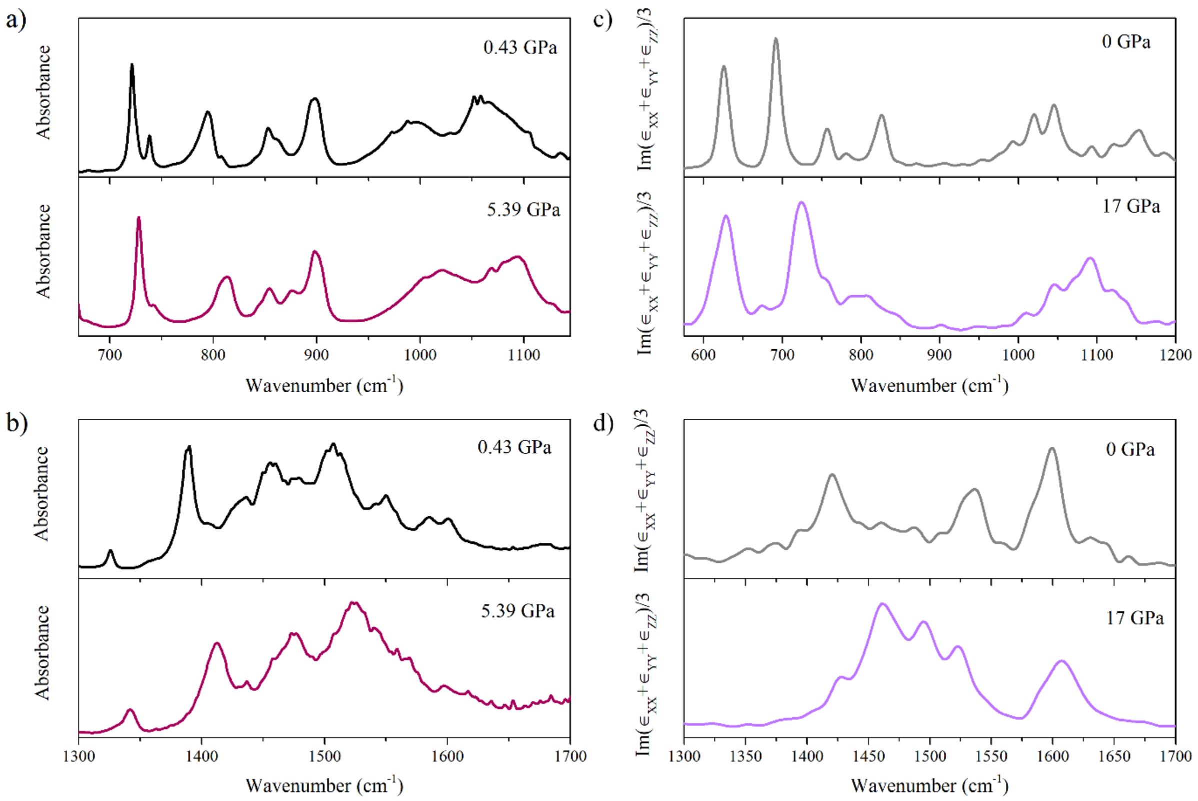

Figure S36. Comparison of experimental at 0.43 and 5.39 GPa (a, b) and calculated at 0 and 17 GPa (c, d) FTIR spectra.

Literature

(1) Zięba, S.; Dubis, A.; Ławniczak, P.; Gzella, A.; Pogorzelec-Glaser, K.; Łapiński, A. Effect of Counter Ions on Physical Properties of Imidazole-Based Proton Conductors. *Electrochim. Acta* **2019**, *306*, 575–589. https://doi.org/10.1016/j.electacta.2019.03.125.

(2) Zhao, J.; Ross, N. L. Non-Hydrostatic Behavior of KBr as a Pressure Medium in Diamond Anvil Cells up to 5.63 GPa. *J. Phys.: Condens. Matter.* **2015**, *27* (18), 185402. https://doi.org/10.1088/0953-8984/27/18/185402.

(3) Piermarini, G. J.; Block, S.; Barnett, J. D.; Forman, R. A. Calibration of the Pressure Dependence of the *R* 1 Ruby Fluorescence Line to 195 Kbar. *J. Appl. Phys.* **1975**, *46* (6), 2774–2780. https://doi.org/10.1063/1.321957.

(4) Dewaele, A.; Torrent, M.; Loubeyre, P.; Mezouar, M. Compression Curves of Transition Metals in the Mbar Range: Experiments and Projector Augmented-Wave Calculations. *Phys. Rev. B* **2008**, *78* (10), 104102. https://doi.org/10.1103/PhysRevB.78.104102.

(5) Wojdyr, M. *Fityk*: A General-Purpose Peak Fitting Program. *J. Appl. Crystallogr.* **2010**, *43* (5), 1126–1128. https://doi.org/10.1107/S0021889810030499.

(6) Frisch, M. J.; Trucks, G. W.; Schlegel, H. B.; Scuseria, G. E.; Robb, M. A.; Cheeseman, J. R.; Scalmani, G.; Barone, V.; Petersson, G. A.; Nakatsuji, H.; Li, X.; Caricato, M.; Marenich, A.; Bloino, J.; Janesko, B. G.; Gomperts, R.; Mennucci, B.; Hratchian, H. P.; Ortiz, J. V.; Izmaylov, A. F.; Sonnenberg, J. L.; Williams-Young, D.; Ding, F.; Lipparini, F.; Egidi, F.; Goings, J.; Peng, P.; Petrone, A.; Henderson, T.; Ranasinghe, D.; Zakrzewski, V. G.; Gao, J.; Rega, N.; Zheng, G.; Liang, W.; Hada, M.; Ehara, M.; Toyota, K.; Fukuda, R.; Hasegawa, J.; Ishida, M.; Nakajima, T.; Honda, Y.; Kitao, O.; Nakai, H.; Vreven, T.; Throssell, K.; Montgomery, J. A.; Peralta, Jr. J. E.; Ogliaro, F.; Bearpark, M.; Heyd, J. J.; Brothers, E.; Kudin, K. N.; Staroverov, V. N.; Keith, T.; Kobayashi, R.; Normand, J.; Raghavachari, K.; Rendell, A.; Burant, J. C.; Iyengar, S. S.; Tomasi, J.; Cossi, M.; Millam, J. M.; Klene, M.; Adamo, C.; Cammi, R.; Ochterski, J. W.; Martin, R. L.; Morokuma, K.; Farkas, O.; Foresman, J. B.; Fox, D. J. Gaussian, Inc., Wallingford CT, 2016.

(7) Chai, J.-D.; Head-Gordon, M. Long-Range Corrected Hybrid Density Functionals with Damped Atom–Atom Dispersion Corrections. *Phys. Chem. Chem. Phys.* **2008**, *10* (44), 6615. https://doi.org/10.1039/b810189b.

(8) Chai, J.-D.; Head-Gordon, M. Systematic Optimization of Long-Range Corrected Hybrid Density Functionals. *J. Chem. Phys.* **2008**, *128* (8). https://doi.org/10.1063/1.2834918.

(9) Ditchfield, R.; Hehre, W. J.; Pople, J. A. Self-Consistent Molecular-Orbital Methods. IX. An Extended Gaussian-Type Basis for Molecular-Orbital Studies of Organic Molecules. *J. Chem. Phys.* **1971**, *54* (2), 724–728. https://doi.org/10.1063/1.1674902.

(10) O'boyle, N. M.; Tenderholt, A. L.; Langner, K. M. Cclib: A Library for Package-independent Computational Chemistry Algorithms. *J. Comput. Chem.* **2008**, *29* (5), 839–845. https://doi.org/10.1002/jcc.20823.

(11) Bader, R. F. W. Atoms in Molecules. *Acc. Chem. Res.* **1985**, *18* (1), 9–15. https://doi.org/10.1021/ar00109a003.

(12) Keith, T. A.; Frisch, M. J. Subshell Fitting of Relativistic Atomic Core Electron Densities for Use in QTAIM Analyses of ECP-Based Wave Functions. *J. Phys. Chem. A* **2011**, *115* (45), 12879–12894. https://doi.org/10.1021/jp2040086.

(13) Espinosa, E.; Molins, E.; Lecomte, C. Hydrogen Bond Strengths Revealed by Topological Analyses of Experimentally Observed Electron Densities. *Chem. Phys. Lett.* **1998**, *285* (3–4), 170–173. https://doi.org/10.1016/S0009-2614(98)00036-0.

(14) Hjorth Larsen, A.; Jørgen Mortensen, J.; Blomqvist, J.; Castelli, I. E.; Christensen, R.; Dułak, M.; Friis, J.; Groves, M. N.; Hammer, B.; Hargus, C.; Hermes, E. D.; Jennings, P. C.; Bjerre Jensen, P.; Kermode, J.; Kitchin, J. R.; Leonhard Kolsbjerg, E.; Kubal, J.; Kaasbjerg, K.; Lysgaard, S.; Bergmann Maronsson, J.; Maxson, T.; Olsen, T.; Pastewka, L.; Peterson, A.; Rostgaard, C.; Schiøtz, J.; Schütt, O.; Strange, M.; Thygesen, K. S.; Vegge, T.; Vilhelmsen, L.; Walter, M.; Zeng, Z.; Jacobsen, K. W. The Atomic Simulation Environment—a Python Library for Working with Atoms. *J. Phys.: Condens. Matter* **2017**, *29* (27), 273002. https://doi.org/10.1088/1361-648X/aa680e.

(15) Batatia, I.; Lin, C.; Hart, J.; Kasoar, E.; Elena, A. M.; Norwood, S. W.; Wolf, T.; Csányi, G. Cross Learning between Electronic Structure Theories for Unifying Molecular, Surface, and Inorganic Crystal Foundation Force Fields. arXiv 2025. https://doi.org/10.48550/ARXIV.2510.25380.
(16) Batatia, I.; Kovács, D. P.; Simm, G. N. C.; Ortner, C.; Csányi, G. MACE: Higher Order Equivariant Message Passing Neural Networks for Fast and Accurate Force Fields. arXiv 2022. https://doi.org/10.48550/ARXIV.2206.07697.
(17) Friede, M.; Hölzer, C.; Ehlert, S.; Grimme, S. *Dxtb* —An Efficient and Fully Differentiable Framework for Extended Tight-Binding. *J. Chem. Phys.* **2024**, *161* (6), 062501. https://doi.org/10.1063/5.0216715.
(18) Grimme, S.; Ehrlich, S.; Goerigk, L. Effect of the Damping Function in Dispersion Corrected Density Functional Theory. *J. Comput. Chem.* **2011**, *32* (7), 1456–1465. https://doi.org/10.1002/jcc.21759.
(19) Martyna, G. J.; Tobias, D. J.; Klein, M. L. Constant Pressure Molecular Dynamics Algorithms. *J. Chem. Phys.* **1994**, *101* (5), 4177–4189. https://doi.org/10.1063/1.467468.
(20) Https://Github.Com/Bracerino/uMLIP-Interactive/Tree/Main/Examples/OrtIMI.
(21) Lebeda, M.; Drahokoupil, J.; Mazáčová, V.; Vlčák, P. Revealing Interstitial Energetics in Ti-23Nb-0.7Ta-2Zr Gum Metal Base Alloy via Universal Machine Learning Interatomic Potentials. *J. Mater. Res. Technol.* **2026**, *41*, 6766–6774. https://doi.org/10.1016/j.jmrt.2026.02.204.
(22) Etter, M. C. Hydrogen Bonds as Design Elements in Organic Chemistry. *J. Phys. Chem.* **1991**, *95* (12), 4601–4610. https://doi.org/10.1021/j100165a007.
(23) Zięba, S.; Dubis, A. T.; Gzella, A. K.; Ławniczak, P.; Pogorzelec-Glaser, K.; Łapiński, A. Toward a New Type of Proton Conductor Based on Imidazole and Aromatic Acids. *Phys. Chem. Chem. Phys.* **2019**, *21* (31), 17152–17162. https://doi.org/10.1039/C9CP01888C.
(24) Fontaine-Vive, F.; Johnson, M. R.; Kearley, G. J.; Cowan, J. A.; Howard, J. A. K.; Parker, S. F. Phonon Driven Proton Transfer in Crystals with Short Strong Hydrogen Bonds. *J. Chem. Phys.* **2006**, *124*.
(25) Grothe, H.; Lund Myhre, C. E.; Nielsen, C. J. Low-Frequency Raman Spectra of Nitric Acid Hydrates. *J. Phys. Chem. A* **2006**, *110* (1), 171–176. https://doi.org/10.1021/jp055521t.
(26) Tchakoutio Nguetcho, A. S.; Ndjoko, P. B.; Kofane, T. C. Mobility and Conductivity of Ionic and Bonded Defects in Hydrogen-Bonded Chains with Nonlinear Interactions. *Eur. Phys. J. B* **2008**, *62* (1), 7–14. https://doi.org/10.1140/epjb/e2008-00110-5.
(27) Matsui, H.; Shimatani, K.; Ikemoto, Y.; Sasaki, T.; Matsuo, Y. Phonon-Assisted Proton Tunneling in the Hydrogen-Bonded Dimeric Selenates of Cs3H(SeO4)2. *J. Chem. Phys.* **2020**, *152* (15), 154502. https://doi.org/10.1063/1.5145108.
(28) Krasnoholovets, V. V.; Tomchuk, P. M.; Lukyanets, S. P. Proton Transfer and Coherent Phenomena in Molecular Structures with Hydrogen Bonds. In *Advances in Chemical Physics*; Prigogine, I., Rice, S. A., Eds.; Wiley, 2003; Vol. 125, pp 351–548. https://doi.org/10.1002/0471428027.ch5.
(29) Matsui, H.; Iwamoto, K.; Mochizuki, D.; Osada, S.; Asakura, Y.; Kuroda, K. Proton Tunneling in Low Dimensional Cesium Silicate LDS-1. *J. Chem. Phys.* **2015**, *143* (2), 024503. https://doi.org/10.1063/1.4926445.
(30) Yan, T.; Xu, Y.; Xi, D.; Yu, Z.; Ma, L.; Zhang, D.; Jiang, R. In-Situ High Pressure Study of Hydrogen-Bonded Energetic Material N-Nitropyrazole. *Chem. Phys. Lett.* **2024**, *852*, 141501. https://doi.org/10.1016/j.cplett.2024.141501.
(31) Loe, C. M.; Chatterjee, S.; Weakly, R. B.; Khalil, M. Observing Vibronic Coupling in a Strongly Hydrogen Bonded System with Coherent Multidimensional Vibrational–Electronic Spectroscopy. *J. Chem. Phys.* **2024**, *161* (17), 174203. https://doi.org/10.1063/5.0226236.
(32) Zięba, S.; Rusek, M.; Katrusiak, A.; Gzella, A.; Dubis, A. T.; Łapiński, A. Helical Model of Compression and Thermal Expansion. *Sci. Rep.* **2023**, *13* (1), 17398. https://doi.org/10.1038/s41598-023-44467-y.
(33) Bernardino, K.; Ribeiro, M. C. C. Hydrogen-Bonding and Symmetry Breaking in the Protic Ionic Liquid 1-Ethylimidazolium Nitrate. *Vib. Spectrosc.* **2022**, *120*, 103358. https://doi.org/10.1016/j.vibspec.2022.103358.
(34) Fan, J.; Wang, P.; Gao, N. Pressure-Dependent Structure and Electronic Properties of Energetic NTO Crystals Dominated by Hydrogen-Bonding Interactions. *Phys. Chem. Chem. Phys.* **2023**, *25* (20), 14359–14367. https://doi.org/10.1039/D3CP01518A.

(35) Chinnakannu, E.; Sankar, M.; Chandran, S.; Thamotharan, K.; Manickam, S. Crystal Growth, Structural, Optical, Thermal, DFT and Z-Scan Analyses of Imidazolium 3,4-Dinitrobenzoate Crystal. *J. Mol. Struct.* **2023**, *1294*, 136419. https://doi.org/10.1016/j.molstruc.2023.136419.
(36) Ardimas; Pakornchote, T.; Sukmas, W.; Chatraphorn, S.; Clark, S. J.; Bovornratanaraks, T. Phase Transformations and Vibrational Properties of Hybrid Organic–Inorganic Perovskite MAPbI3 Bulk at High Pressure. *Sci. Rep.* **2023**, *13* (1), 16854. https://doi.org/10.1038/s41598-023-43020-1.
(37) *The Hydrogen Bond and the Water Molecule: The Physics and Chemistry of Water, Aqueous and Bio Media*, 1st ed.; Maréchal, Y., Ed.; Elsevier: Amsterdam Boston, 2007.
(38) Spahr, E. J.; Wen, L.; Stavola, M.; Boatner, L. A.; Feldman, L. C.; Tolk, N. H.; Lüpke, G. Proton Tunneling: A Decay Channel of the O-H Stretch Mode inKTaO3. *Phys. Rev. Lett.* **2009**, *102* (7). https://doi.org/10.1103/physrevlett.102.075506.
(39) Fischer, S. F.; Hofacker, G. L.; Sabin, J. R. Proton-Phonon Coupling in a Hydrogen Bonded System. *Phys. Kondens. Materie.* **1969**, *8* (4), 268–278. https://doi.org/10.1007/BF02422703.
(40) Zięba, S.; Gzella, A.; Dubis, A. T.; Łapiński, A. Combination of Negative, Positive, and Near-Zero Thermal Expansion in Bis(Imidazolium) Terephthalate with a Helical Hydrogen-Bonded Network. *Cryst. Growth Des.* **2021**, *21* (7), 3838–3849. https://doi.org/10.1021/acs.cgd.1c00167.
(41) Fumino, K.; Peppel, T.; Geppert-Rybczyńska, M.; Zaitsau, D. H.; Lehmann, J. K.; Verevkin, S. P.; Köckerling, M.; Ludwig, R. The Influence of Hydrogen Bonding on the Physical Properties of Ionic Liquids. *Phys. Chem. Chem. Phys.* **2011**, *13* (31), 14064. https://doi.org/10.1039/c1cp20732f.
(42) Nakamoto, K.; Margoshes, M.; Rundle, R. E. Stretching Frequencies as a Function of Distances in Hydrogen Bonds. *J. Am. Chem. Soc.* **1955**, *77* (24), 6480–6486. https://doi.org/10.1021/ja01629a013.
(43) Zięba, S.; Piotrowska, A.; Mizera, A.; Ławniczak, P.; Markiewicz, K. H.; Gzella, A.; Dubis, A. T.; Łapiński, A. Spectroscopic and Structural Study of a New Conducting Pyrazolium Salt. *Molecules* **2021**, *26* (15), 4657. https://doi.org/10.3390/molecules26154657.
